# New insights into tuberous sclerosis complex: from structure to pathogenesis

**DOI:** 10.3389/fcell.2025.1595867

**Published:** 2025-06-27

**Authors:** Chao-Sheng Chen, Christopher H. S. Aylett

**Affiliations:** Section for Structural and Synthetic Biology, Department of Infectious Disease, Imperial College London, London, United Kingdom

**Keywords:** GAP, hamartin, mTORC1, rapamycin, RHEB, TBC1D7, TSC, tuberin

## Abstract

Tuberous sclerosis complex is a genetic disorder characterised by the formation of benign tumours in multiple organs, primarily due to pathogenic variants in the *TSC1* and *TSC2* tumour suppressor genes. These genes encode hamartin and tuberin, respectively, which together with TBC1D7 form a crucial protein complex regulating cell growth and proliferation through mTOR signalling and other pathways. This review provides an overview of recent progress in understanding the molecular structure and function of this key protein complex, its role in cellular processes, pathogenesis, and current and future therapeutic strategies.

## Introduction: the history of tuberous sclerosis complex

Tuberous Sclerosis Complex (TSC, Orphanet: 805, OMIM entry: 191,100) is an autosomal dominant disorder with an incidence of approximately 1 in 6,000–10,000 live births (Northrup et al., 2021). All age and ethnic groups from both genders can be affected by TSC (Northrup et al., 2021; Randle, 2017; Brigo et al., 2018; Osborne et al., 1991). The study of TSC is ongoing and has spanned almost 190 years. The earliest known report is an illustration published by Pierre François Olive Rayer in 1835 (Rayer, 1835) of a patient’s face dotted with various small erythematous papules that resemble the “facial angiofibromas” of TSC. The first pathological description of the lesions of TSC identified in two different organs was documented around 30 years later by Friedrich Daniel von Recklinghausen (von Recklinghausen, 1862). In 1880, the first detailed description of the cerebral pathology of TSC was reported by Desire-Magloire Bourneville (Bourneville, 1880; Bourneville and Brissaud, 1881). Five years later, Balzer and Menetrier reported TSC in a mother and daughter and first linked the characteristic facial angiofibromas with TSC (Balzer and Menetrier, 1885). In 1908, Heinrich Vogt proposed quasi-diagnostic criteria for TSC: a triad consisting of epilepsy, “idiocy,” and the aforementioned facial angiofibromas (Vogt, 1908). Most individuals who manifest all three features of Vogt’s triad will have TSC; this is because facial angiofibromas have a high level of specificity for the disease. However, the fact that Vogt’s triad would likely fail to diagnose roughly half of the TSC patients that we now recognise implies that the clinical manifestations can differ widely. In 1979, the first edition of Tuberous Sclerosis edited by Manuel Gomez was published. It was the first comprehensive overview of TSC with contributions from experts from different disciplines (Gomez, 1979), covering most aspects of the disease and recognising that TSC manifestations extended beyond a single clinical discipline.

In 1975, dissatisfied by the lack of understanding of their children’s disease and the lack of ongoing research, Adrianne Cohen, Susan Diaz, Linda Hamm, and Verna Morris, each a mother of a child affected by TSC, established the National Tuberous Sclerosis Association, later renamed the Tuberous Sclerosis Alliance in 2000. The Tuberous Sclerosis Alliance has grown into a successful support organization, providing information for professionals and families, supporting clinical and fundamental research, as well as TSC patients, their families and broader TSC communities. Many of the more recent major advances in TSC research would have been much more difficult without the funding provided by these TSC related organisations in addition to that provided by governments and other charities around the world.

## Clinical features of TSC

TSC leads to the growth of non-malignant tumours and structural abnormalities in development, both affecting multiple organs (Curatolo et al., 2008; Roach, 2016; Henske et al., 2016). The clinical features of TSC are highly variable and the distribution of lesions, typically within the brain, heart, lung, kidney and skin, although this list is not exhaustive, is unpredictable. Epilepsy is the most common symptom, affecting 80%–90% of patients with TSC (Holmes et al., 2007). Subependymal giant cell astrocytomas (SEGAs), occur in 10%–15% of TSC patients, often causing TSC-related morbidity and occasionally mortality (Henske et al., 2016). Cognitive and neurobehavioural issues are common in TSC. Approximately 50% of individuals with TSC have some degree of intellectual disability, while at least two-thirds of individuals with the disorder struggle with TSC-associated neuropsychiatric disorders (TANDs) (Joinson et al., 2003; de Vries et al., 2015).

Lymphangioleiomyomatosis (LAM) is the primary pulmonary manifestation of TSC which causes cystic lung destruction, pneumothorax (lung collapse), and chylous pleural effusion (Henske and McCormack, 2012). LAM rarely, if ever, occurs in males. Asymptomatic LAM (as defined by the presence of multiple lung cysts) occurs in up to 80% of women with TSC. Symptomatic LAM occurs in ∼5–10% of women with TSC and often leads to respiratory failure (Henske and McCormack, 2012). LAM tends to progress more rapidly in premenopausal women than in postmenopausal women; the level of oestrogen has a strong influence on the development of LAM, and the disease often stabilizes after menopause (Sullivan, 1998). Multifocal micronodular pneumocyte hyperplasia (MMPH) can occur in both genders with TSC and is usually asymptomatic (von Ranke et al., 2015). Renal angiomyolipomas (AMLs) and cysts are the two most common renal lesions in TSC, which can be detected from early childhood, while up to 67% of patients with TSC have AMLs at autopsy (Bernstein, 1991). Rarer manifestations include renal cell carcinoma, the epithelioid variant of AML and oncocytoma (Bissler and Kingswood, 2004). Almost all TSC patients develop skin manifestations (Teng et al., 2014). These include facial angiofibromas, ungual fibromas, fibrous cephalic plaques, shagreen patches, and focal hypopigmentation changes. These dermatologic symptoms emerge at different time points and can help in making a non-invasive clinical diagnosis of TSC (Teng et al., 2014). The disease exhibits significant phenotypic variability, and there are hundreds of possible unique presentations and courses of the illness which are influenced by the location, penetrance, and the severity of hamartoma formation. The heterogeneous nature of the condition together with the range of tissues it can affect make TSC a particularly challenging disease both to diagnose and to treat (Roach, 2016; Henske et al., 2016).

Genetic linkage studies in families segregating TSC identified locus heterogeneity with loci on chromosomes 9 and 16 leading to apparently indistinguishable phenotypes (Fryer et al., 1987; Kandt et al., 1992). Using gene mapping, combined with the known genetic information collected from the linkage studies, it was possible to identify germ-line loss-of-function variants in *TSC1* (chromosome 9q34; hg38: g.9:132891348-132946874; NM_000368.5; 23 exons) and *TSC2* (chromosome 16p13.3; hg38: g.16:2047985-2089491; NM_000548.5; 42 exons) as the primary cause of TSC (van Slegtenhorst et al., 1997; European Chromosome 16 Tuberous Sclerosis Consortium, 1993). TSC follows the Knudson “two-hit” tumour-suppressor gene model (Knudson, , 1971); that is, a germline alteration either occurring *de novo* in the zygote or inherited from an affected parent, and inactivating one allele of TSC1 or TSC2, is complemented by a second somatic alteration, often loss of heterozygosity (LOH), in the remaining wild-type allele. TSC is a lifelong condition with no currently available cure, however, many clinical manifestations can now be monitored and managed, including seizures, TAND, skin lesions, and lung (LAM) and kidney issues (Stuart et al., 2021).

## Diagnosis of tuberous sclerosis complex

Around ∼70% of clinically diagnosed TSC patients have *TSC2* pathogenic variants, while 20% carry *TSC1* pathogenic variants (Sancak et al., 2005). Some of the remaining ∼10% of TSC individuals are classified as having “no mutation identified” (NMI) despite thorough conventional molecular diagnostic assessment such as exon-based sequencing and analysis for large genomic deletions in *TSC1* and *TSC2*. Several possible factors contribute to NMI status in TSC patients, including mutation detection failure due to technical issues, unknown epigenetic modifications that may affect TSC gene expression, mosaicism and the occurrence of pathogenic variants deep within introns. Mosaicism occurs when a mutation in *TSC1* or *TSC2* arises during (early) embryonal development so that only a proportion of cells in the body carry the causative variant (Sancak et al., 2005; Nellist et al., 2015; Tyburczy et al., 2015). Next generation sequencing (NGS) technology has improved molecular diagnostics for individuals with TSC (Metzker, 2010), helping to reduce uncertainty and anxiety and provide TSC patients with greater confidence in their understanding of their condition. Better diagnosis can lead to improved monitoring and management of the disease and thereby save both patients and resources (Nellist et al., 2015; Tyburczy et al., 2015).

On the whole, individuals with a pathogenic *TSC2* variant are likely to present with more severe manifestations of the disease than those with a pathogenic *TSC1* variant. A milder phenotype is also observed in individuals with NMI; although considerable phenotypic variability still occurs (Nellist et al., 2015). According to the *TSC1* and *TSC2* Leiden Open Variant Databases (LOVD) (Fokkema et al., 2021), there are currently more than 6,000 *TSC1* and *TSC2* variants identified in TSC patients, with over 1,400 unique variants in *TSC1* and more than 4,900 in *TSC2*.

Most pathogenic *TSC1* variants are inactivating due to truncation of the open reading frame (ORF), with missense variants accounting for <5% of pathogenic variants identified to date. In contrast, ∼20% of likely pathogenic *TSC2* variants are missense changes. However, many variants identified in *TSC1* and *TSC2* are referred to as variants of uncertain clinical significance (VUS) (Mozaffari et al., 2009; Hoogeveen-Westerveld et al., 2011; Hoogeveen-Westerveld et al., 2012; Hoogeveen-Westerveld et al., 2013; Wentink et al., 2012): there is insufficient data to conclude whether these variants are either benign or pathogenic. The recent advances in resolving the molecular structure of the TSC protein complex (TSCC), and insights deduced from this structural information will aid clinicians in advising on the impact of different variants on TSCC function, and their likely pathogenicity (Ramlaul et al., 2021; Yang et al., 2021; Bayly-Jones et al., 2024).

## Canonical signalling mechanism of the TSCC

**FIGURE 1:**
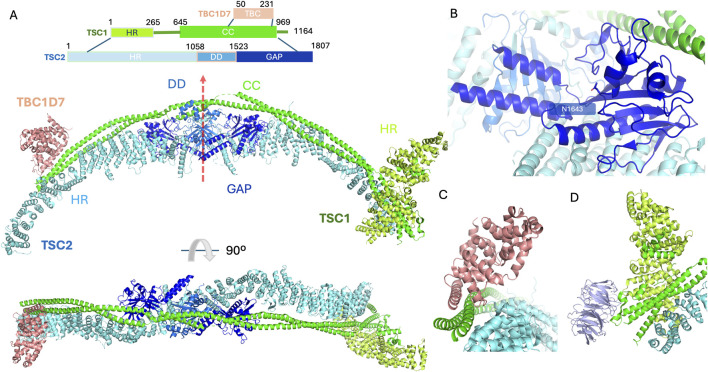
The structure of the TSCC (PDB code 9ce3). **(A)** Primary domain structure schematic illustrating individual domains on TSC subunits. The elongated structure contains 2 copies of TSC1 and 2 copies of TSC2 with a single TBC1D7 bound at the C-terminus of TSC1. The domains are coloured as follows, for TSC1, the heat repeat domain (HR) and coiled-coil domain (CC) are coloured limon and green, respectively; for TSC2, the heat repeat domain (HR), dimerisation domain (DD) and GAP domains are coloured cyan, marine and blue, respectively. The TBC1D7 is coloured light pink. 2 TSC2 subunits sit back-to-back via their DDs with the pseudo-C2 axis shown by a red arrow. The 2 TSC1 subunits form a long coiled-coil along the 2 TSC2 subunits. The GAP domain important for TSCC function is accessible. The N-terminus of TSC1 clamped on one end of the TSC2 stabilises the TSCC. The C-termini of the TSC1 dimer also contribute to maintaining the intact TSCC. **(B)** Close-up view of the GAP domain of the TSC2 subunit. The catalytic pocket is surrounded by the TSC1 coiled-coil region and the DD from the same subunit with essential residue N1643 highlighted in stick representation accessible to incoming molecules. **(C)** Close-up view of helix-helix interactions between TBC1D7 and the TSC1 dimer along on one of the TSC2 subunit. **(D)** Close-up view of WIPI3 interactions with the TSC1, WIPI3 coloured in light purple. N-terminal HR domain from individual TSC1 subunits dimerise and clamp on to the end of one of the TSC2 heat repeat domains. WIPI3 binds to the very tip of the complex through a conserved motif in TSC1.

**FIGURE 2:**
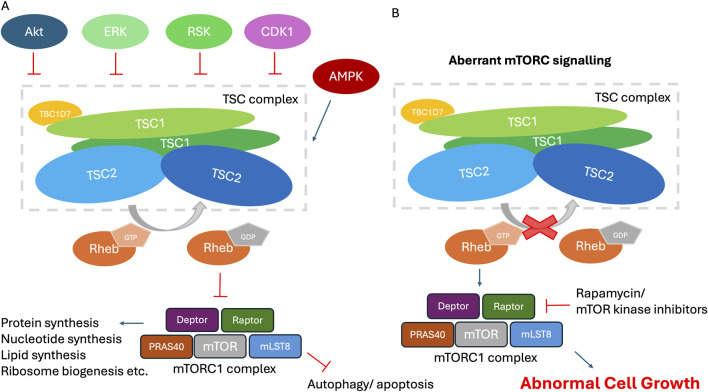
The role of the TSCC in the mTORC1 signaling pathway. **(A)** The TSCC, selected upstream regulators and downstream effectors. The TSCC is shown comprising a pseudo-symmetrical TSC2 dimer (blue) interacting with two intertwined TSC1 molecules (green), and one TBC1D7 molecule (orange) bound to the one end of TSC1 dimer. Selected mTORC1 upstream-input signaling and downstream-output signaling pathways and their physiological effects are shown. **(B)** Cells with inactivating mutations within *TSC1* or *TSC2* possess constitutively activated RHEB. RHEB activates the mTORC1 signaling network, leading to increased protein translation and cell growth on the one hand and decreased autophagy and apoptosis on the other hand, among many other effects, all of which results in abnormal cell growth.

*TSC1* encodes a ∼130 kDa (1,164 amino acid) protein known as TSC1, or hamartin. TSC1 consists of an N-terminal α-helical HEAT repeat domain and a large (∼350 Å) coiled-coil forming helix spanning residues 645–969. *TSC2* encodes a ∼200 kDa (1,807 amino acid) protein known as TSC2, or tuberin. TSC2 consists almost entirely of α-solenoid, with a catalytic GTPase-activating protein (GAP) domain close to the C-terminus (residues 1,523–1,807) that is specific for the GTPase Ras homologue enriched in brain (RHEB). TSC1 and TSC2, together with the small globular third subunit Tre2-Bub2-Cdc16-1 domain family member 7 (TBC1D7; 293 amino acids, ∼34 kDa), form the TSCC (Figure 1A) (Dibble et al., 2012), which negatively regulates the mTORC1 pathway, a central regulator of cell growth and metabolism (Figure 2A). The TSCC serves as a key inhibitory hub for this master kinase and loss of any of the three components of the TSCC can cause inappropriate, growth-factor-independent mTORC1 activity (Figure 2B). The mTORC1 complex is formed by the mTOR protein kinase and other 4 protein subunits that control the balance between anabolic and catabolic processes in cells (Figure 2A). Its activation occurs in response to a series of stimuli relevant to cell growth, including nutrient availability, growth factor signals and stress, and it is the nexus regulating much of the cell’s biosynthetic activity, from the manufacture of proteins to lipids, to recycling through autophagy (Ramlaul and Aylett, 2018).

## The molecular structure of the TSCC

The TSCC forms an elongated scorpion-like architecture with a 2:2:1 stoichiometry of TSC1, TSC2 and TBC1D7 (Figure 1A) (Ramlaul et al., 2021; Yang et al., 2021; Bayly-Jones et al., 2024). The body of the TSCC exhibits pseudo-C2 symmetry; TSC2 forms an extended, α-solenoid dimeric scaffold with approximate twofold rotational (C2) symmetry (red arrow in Figure 1A). The GAP domain of each TSC2 subunit is located next to the dimerization interface (Figure 1A). TSC1 consists of an α-helical HEAT repeat domain located in the N-terminus and a long coiled-coil to the very end of the C-terminus; the TSC1 coiled-coil domain is asymmetric within the TSCC which breaks the C2 symmetry of the TSC2 dimer. All protein sequence numbering is based on the numbering used for human species in UniProt (https://www.uniprot.org/).

Together the central regions of each TSC1 subunit form an extended, ∼350 Å long, dimeric coiled-coil structure (residues 645–969) that runs along the crest of the TSC2 dimer (Figure 1A), contacting multiple HEAT repeats on both TSC2 subunits. The TSC1-TSC2 interface (1,761 Å^2^) is likely to contribute to the stability of the entire TSCC (Yang et al., 2021). In addition, a large buried surface area (2,805 Å^2^) has been identified between the 2 TSC2 subunits (residues 1,058–1,523). The large interface areas indicate relatively strong interactions, and suggest that the TSC2 dimer has the capacity to form independently of TSC1 (Yang et al., 2021). The GAP domains of TSC2 symmetrically abut the core module on each side of the TSC2 dimerisation domain. This catalytic domain contains a 7-stranded β-sheet stabilised by its neighbouring long α-helix. TSC1 coiled-coils also make contact with the GAP domains from both TSC2 subunits (Figure 1B). The catalytic pockets open outwards and are poised to bind RHEB, however, the molecular details of exactly how RHEB interacts with the TSCC remain elusive and await further investigation (Ramlaul et al., 2021; Yang et al., 2021; Bayly-Jones et al., 2024).

The pair of TSC1 proteins form a dimer. TBC1D7 utilises a helical region (residues 70–95) to anchor itself upon the C-terminal segment (residues 937–971) of the TSC1 dimeric coiled-coil via helix-helix interactions, contacting both TSC1 subunits, but making little-to-no contact with TSC2 (Figure 1C). The presence of the N-terminus of TSC2 prevents the possibility of a second TBC1D7 becoming bound. The two HEAT repeats, one from each TSC1 molecule, at the N-termini of the dimeric TSC1 complex form a large dimer clamped at the opposite tip of the TSCC from the TBC1D7 subunit. In addition to the HEAT repeats, a central pseudo-symmetrical hydrophobic interface is formed by residues 197–237, and the N-terminal TSC1 dimer is connected to the TSC1 coiled-coil by extensive intrinsically disordered loop regions. One of the dimerised TSC1 HEAT repeat domains engages asymmetrically with the dimeric TSC1 coiled-coil region (Figure 1D), sandwiching it onto the end of one of the TSC2 N-termini. Additionally, this same engaged TSC1 subunit can interact with WD repeat domain phosphoinositide interacting protein 3 (WIPI3) through a conserved motif (Figure 1D) (Bayly-Jones et al., 2024).

TSCC negatively regulates mTORC1 by functioning as a GAP towards RHEB. RHEB is one of the Ras-related small G proteins lying directly upstream of mTORC1, which is localised to the endomembrane system via C-terminal farnesylation (González and Hall, 2017). Ras-related GTPases can bind guanosine triphosphate (GTP) and guanosine diphosphate (GDP), and the type of nucleotide bound is coupled to a conformational change. Typically, binding to GTP locks them in an active conformation that is recognised by effector proteins, while there is no interaction with the effectors in the inactive GDP bound state (Hansmann et al., 2020). The TSCC localizes to the lysosome when none of its suppressive inputs are present to convert active GTP-bound RHEB-GTP to the inactive GDP-bound form. It is likely that TSCC recruitment involves multiple factors that are partially redundant and context-dependent (Demetriades et al., 2014; Liu et al., 2018). RHEB binding is essential for the TSCC to become anchored at the lysosomal surface (Menon et al., 2014).

Guanine nucleotide exchange factors (GEFs) stabilize the nucleotide-free form of GTPases to allow release of the bound nucleotide. GEF-mediated nucleotide release and GTPase-GEF dissociation is followed by binding of GTP, since the GTP concentration in cells is much higher than that of GDP. Ras GTPases have a highly conserved glutamine residue lending intrinsic GTP hydrolysis activity. However, because of incomplete active sites, ras-related GTPases, and RHEB in particular, exhibit extremely low innate enzymatic activity, lacking a catalytic glutamine residue for intrinsic or GAP-stimulated GTP hydrolysis (Marshall et al., 2009). GAPs interact with GTPases and contribute catalytic site residues to stimulate GTP hydrolysis and thus effector inactivation (Calixto et al., 2019). TSC2 catalyses the hydrolysis of the active GTP-bound RHEB via an asparagine-thumb mechanism to yield its inactive GDP-bound state (Hansmann et al., 2020; Marshall et al., 2009; Calixto et al., 2019). RHEB has remained without an identifiable GEF for a considerable time period, and may not require one due to its low intrinsic GTPase activity, however recent work has suggested that non-canonical GEF activity via the lysosomal factor ATP6AP1 (Feng et al., 2024), or via a yet to be established mechanism through the epidermal growth factor receptor (EGFR) pathway (He et al., 2025), may contribute to conversion of RHEB into the active GTP-bound form. While in the presence of growth factors, mTORC1 can be activated via signalling pathways including the class I phosphatidylinositol-3-kinase (PI3K) and its downstream serine/threonine kinase protein kinase B (AKT), which directly phosphorylates 5 serine and threonine residues on TSC2. The phosphorylated TSCC dissociates from the lysosomal surface into the cytosol, relieving TSCC-mediated inhibition of lysosomal GTP-RHEB, thereby allowing RHEB-GTP to accumulate and trigger mTORC1 signalling (Menon et al., 2014; Huang and Manning, 2008; Inoki et al., 2002).

## TSC2 regulation by phosphorylation

TSC2 has long been understood to be the key TSC factor modulating mTORC1 signalling as it contains the only enzymatic domain within the TSCC, acting as a GAP for RHEB (Figure 1B). The function of TSC2 GAP activity is regulated by many factors including insulin, energy stress oxygen pathways, growth factors, etc., (Ramlaul and Aylett, 2018; González and Hall, 2017; Demetriades et al., 2014; Huang and Manning, 2008), which are generally achieved through posttranslational modifications such as phosphorylation (Supplementary Table S1). Multiple signalling kinases in turn stimulate (AKT, ERK, and RSK-1) (Inoki et al., 2002; Ma et al., 2005; Roux et al., 2004) or inhibit (AMPK and GSK-3β) (Inoki et al., 2006) mTORC1 activity via phosphorylation of specific sites on TSC2 (Figure 2A). In the presence of insulin and various growth factors, AKT directly phosphorylates TSC2 at a common recognition motif, RxRxxS/T (R, arginine; S, serine; T, threonine; x, any amino acid), that is located at multiple sites, including S939, S981, S1130, S1132 and T1462. Insulin-induced dissociation of the TSCC from the lysosome and subsequent RHEB-dependent activation of mTORC1 requires the phosphorylation of multiple sites on TSC2 by AKT, thereby providing a mechanism to negatively regulate the TSCC by the PI3K-AKT pathway (Inoki et al., 2002; Cai et al., 2006). Phosphorylation of TSC2 at serine residues 939 and 981 does not alter its intrinsic GAP activity toward RHEB, instead, spatially releasing TSC2 from the lysosome, followed by 14-3-3 protein binding of TSC2 which sequesters it in the cytosol. Thus, TSC2 bound by 14-3-3 in response to AKT phosphorylation is sequestered away from its target GTPase, RHEB, which is localised to the endosomal system via its farnesylation, relieving the growth inhibitory effects of the TSCC (Inoki et al., 2002; Cai et al., 2006). The mitogen-activated protein kinase (MAPK)-activated kinase, p90 ribosomal S6 kinase 1 (RSK1), was also found to interact with and phosphorylate TSC2 at S1798 and S1364 (Roux et al., 2004; Ballif et al., 2005). When RSK1 phosphorylates TSC2, it similarly results in translocation of the TSCC to the cytoplasm, leading to increased mTORC1activation, and ultimately cell growth. It is notable that several functional isoforms of TSC2, expressed in multiple tissues, lack residues 946-988 (isoform 4) and/or residues 1,272–1,294 (isoforms 4 and 5). These exons are located on loop 1 (917–1,020) and loop 3 (1,226–1,496). Given the importance of these regions for phosphorylation dependent regulation, these isoforms might be expected to exhibit quite distinct functionality. However given the incomplete understanding of the mechanism of release from the lysosome, this remains an area of active investigation.

Activation of the canonical Wnt pathway inhibits GSK3, resulting in hypophosphorylation and thus stabilization of β-catenin, which translocates into the nucleus and forms a complex with the DNA binding protein T cell factor (TCF). β-catenin serves as an essential coactivator through recruiting enzymes such as creb binding protein (CBP) that promotes chromatin remodelling and transcriptional initiation/elongation (Moon et al., 2004). Wnt signalling is also known to stimulate translation and cell growth via the TSCC-mTORC1 pathway (Inoki et al., 2006). Through inhibition of GSK3, mTORC1 can be activated by Wnt signalling. GSK3 inhibits the mTORC1 pathway by phosphorylating both S1337 and S1341 of TSC2 in a manner dependent on AMPK priming phosphorylation on S1346. AMPK, a protein kinase activated by AMP, is a cellular energy sensor and plays an important role in cellular energy homeostasis. AMPK directly phosphorylates TSC2, and the AMPK-dependent phosphorylation of TSC2 is critical for the coordination between cell growth and cellular energy levels (Inoki et al., 2006).

Inhibition of protein synthesis results in rapid activation of mTORC1 signalling, this is due to a feedback loop between mTORC1 and the translation machinery (Zhan et al., 2019). For this pathway, TSCC is required for mTORC1 activation but independently of AKT. Kinase activity of protein kinase c delta (PKC-δ) has been identified as crucial for such mTORC1 activation. PKC-δ can phosphorylate and inactivate TSC, leading to mTORC1 activation. Two serine residues, S932 and S939, on TSC2 have been reported to be phosphorylated by PKC-δ in response to translation inhibition. The precise nature of the upstream signal that activates PKC-δ remains elusive. The physiological significance of this compensatory feedback loop is to maintain translational homeostasis in cells, thereby preventing potential cell death (Zhan et al., 2019).

## Newly uncovered kinases also target TSC2 to regulate mTORC1 signalling

In addition to the well-studied kinases mentioned above, recent work has highlighted similar patterns of regulation through previously uncharacterised pathways. The novel kinase dual-specificity tyrosine phosphorylation-regulated kinase 1 A (DRYK1A), upon interaction with the TSCC, regulates mTORC1 activity and cell size (Wang et al., 2024). DRYK1A is a ubiquitously expressed kinase belonging to the CMGC group (Cyclin-dependent kinases, Mitogen-activated protein kinase, Glycogen synthase kinases, and CDK-like kinases group). Inactivating variants in DRYK1A are associated with microcephaly. DRYK1A is involved in numerous cellular processes such as the cell cycle, microtubule assembly, and vesicle trafficking (Arbones et al., 2019). DYRK1A can bind TSC1 via its own kinase domain and phosphorylate TSC2 at T1462. This modification inhibits TSC activity and promotes mTORC1 signalling. Similar results are observed in cells originating from different species including human, mouse and *Drosophila melanogaster* cells, suggesting that DYRK1A-mediated regulation of mTORC1 activity is a conserved mechanism to regulate cell size and development (Wang et al., 2024).

TSC2 has also now been shown to be phosphorylated by death associated protein kinase 1 (DAPK1) at Ser939 (Stevens et al., 2009; Wei et al., 2021a). DAPK1 belongs to a calmodulin (CaM)-regulated death associated protein-like serine/threonine kinase superfamily, and plays a central role in a diverse range of signal transduction pathways, such as growth factor activation, apoptosis, and autophagy (Shohat et al., 2002). It comprises an N-terminal kinase domain, followed by an autoregulatory Ca^2+^/CaM-binding domain that regulates the catalytic activity by binding to the catalytic cleft and functioning as a pseudosubstrate. Autophosphorylation of DAPK1 at Ser308 interferes with CaM binding and blocks DAPK catalytic activity (Shohat et al., 2002). DAPK1 is activated by the binding of Ca^2+^-activated CaM to the autoregulatory/CaM-binding domain, exposing the catalytic site of the kinase. The interaction between CaM and DAPK1 is enhanced by the dephosphorylation of Ser308, which serves as a marker for DAPK1 activation. DAPK1 is dephosphorylated by calcineurin and activated DAPK1 interacts with TSC2 via its death domain and phosphorylates TSC2, resulting in mTORC1 activation (Shohat et al., 2002).

Finally, in the heart, or in isolated cardiomyocytes or fibroblasts, protein kinase G1 (PKG1) phosphorylates two adjacent serine residues S1364 and S1365 in TSC2 (Ranek et al., 2019). PKG1 is essential for protection against heart disease, acting as a key player for nitric oxide and natriuretic peptide signalling (Kim and Kass, 2016). PKG1 phosphorylation or phosphorylation-resistant or mimetic variants of these 2 serine residues on TSC2 can bidirectionally regulate mTORC1 activity stimulated by growth factors or haemodynamic stress, which leads to modulation of cell growth and autophagy (Ranek et al., 2019). In cardiomyocytes and the myocardium, pathological stress triggers TSC2 phosphorylation along with activation of mTORC1, whereas blocking this phosphorylation by alanine mutation for the PKG1-modified serines intensified the pathology, supporting a role for the phosphorylation of TSC2 at this site as a negative feedback loop regulating mTORC1 activity. On the other hand, substituting serine with glutamic acid induced the protective outcomes or the effects of more selective phosphorylation by PKG1, supporting this idea further. TSC2, mTOR and related mTOR-complex proteins are ubiquitously expressed in mammalian cells, and this is also the case for PKG1 expression in many cell types. Therefore, there are promising potential therapeutic roles for PKG1 activators in diseases such as TSC in which altered mTORC1 signalling is the issue, not only limited to the heart.

## Kinase-independent regulation of TSC2

Apart from phosphorylation, TSC2 recently has been shown to interact with protein arginine methyltransferase 1 (PRMT1) and to be methylated at R1457 and R1459 (Gen et al., 2020). Protein arginine methyltransferases (PRMTs) catalyse the addition of methyl residues from S-adenosyl methionine (SAM) to guanidino nitrogen atoms of arginine residues (Morales et al., 2016). PRMT1 is ubiquitously expressed and preferentially recognises glycine-arginine rich (GAR) motifs (RGG/RG motif) on a target protein, and it is a major methyltransferase that is linked to metabolic disorders as well as cancer development (Blanc and Richard, 2017). The methylation status of the TSC2 protein blocks AKT phosphorylation at T1462 and any other affected residues, preventing the suppression of TSC activity via such phosphorylation.

More recent results, which require further confirmation, have suggested that intracellular calcium ion (Ca^2+^) concentration modulates the mTORC1 pathway via binding of the Ca^2+^ sensor protein calmodulin to TSC2 directly (Amemiya et al., 2024). CaM may disrupt the binding of TSC2 to RHEB in a Ca^2+^-dependent manner, promoting the dissociation of TSC2 from lysosomes without affecting AKT-dependent phosphorylation of TSC2. This implies that the regulatory mechanism of TSC2 by Ca^2+^/CaM may be distinct from the previously established mechanism of action of TSC2 and further efforts are needed to investigate the role of calcium ion and CaM involvement in the TSC-mTORC 1 pathway (Amemiya et al., 2024).

The molecular mechanisms anchoring RHEB and mTORC1 at lysosomes are well investigated (Kim and Guan, 2019; Condon and Sabatini, 2019). Details underlying the recruitment of the TSCC and associated factors to lysosomes have only begun emerging more recently (Demetriades et al., 2016; Prentzell et al., 2021). While most studies have highlighted the role of TSC1 and accessory proteins in lysosomal recruitment, recent results have suggested a possible role for TSC2 specifically in the linkage between lysosomes and stress granules (SGs). Ras GTPase-activating protein-binding proteins (G3BP1 and G3BP2) are widely recognised as RNA-binding proteins forming the core components of SGs. Surprisingly, G3BPs reside at the cytoplasmic surface of lysosomes; G3BP1 but not G3BP2 has been suggested to act in a non-redundant manner to tether the TSCC to lysosomes and suppress activation of mTORC1. The C-terminal domains of G3BP1, harbouring the RNA recognition motif (RRM), and the arginine-glycine rich (RGG) repeats, has been suggested to be involved in binding to TSC2. Moreover, the N-terminal NTF2L domain of G3BP1 can also interact with lysosomal associated membrane proteins (LAMPs), bridging TSC2 to LAMP proteins (Prentzell et al., 2021). Interestingly, and in a similar vein, TSC2 has been suggested to physically interact with high-density lipoprotein binding protein (HDLBP), an mRNA binding protein (KH domain family) known as vigilin, which is another core stress granule protein (Kosmas et al., 2021). SGs contain translation initiation complexes and mRNAs, serving a cytoprotective role regulating gene expression by sequestering specific transcripts (Kedersha and Anderson, 2007). Vigilin has 14 hnRNP/KH (heterogeneous nuclear ribonucleoprotein/K homology) domains, is involved in multiple cellular processes including ribosome association and heterochromatin regulation, and is known to be upregulated in cancer. Early yeast two-hybrid screens suggest vigilin interacts with TSC2 via its first 6 hnRNP homology (KH) domains (Cheng and Jansen, 2017; Woo et al., 2011). Vigilin has been shown to recruit TSC2 to SGs upon oxidative and thermal stress, colocalizing with a SG marker G3BP1, while knocking down vigilin reduced translocation of TSC2 to SGs. TSC2-deficient cells also have an increased number of SGs under oxidative/thermal stress. While these reported interactions of TSC2 with two separate core SG factors strengthen these results, this possible relationship between SGs and TSCC activity is still emerging and requires further investigation to confirm that these associations are not dependent upon other lysosomal factors.

## Proposed physiological roles for TBC1D7

**FIGURE 3:**
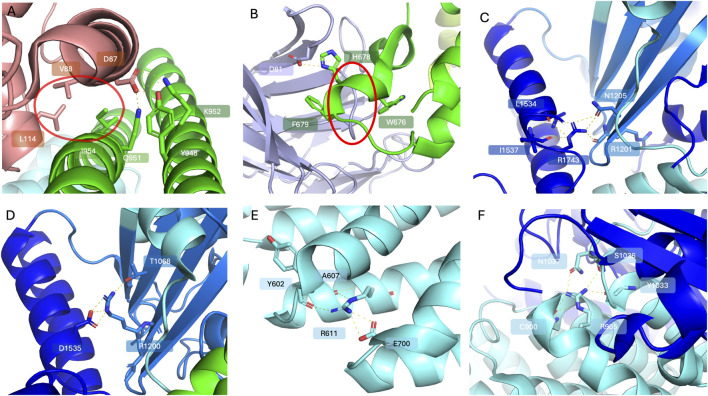
Selected frequent TSC variants (PDB code 9ce3). The TSC1 subunits are coloured limon and green; the TSC2 subunits are coloured blue and cyan; TBC1D7 is coloured light pink. Highlighted residues are shown in stick representation with oxygen in red and nitrogen in blue. **(A)** A close-up view of the interacting patches between TSC1 and TBC1D7; D87 of TBC1D7 forms a polar interaction network with Q951, K952 and Y948 of TSC1, while a hydrophobic interaction patch circled in red can be observed between V88 and L114 of TBC1D7 and I954 of TSC1. **(B)** WIPI3 interactions with the TSC1. WIPI3 coloured in light purple. D81 of WIPI3 forms polar interactions with H678 of TSC1. W676 and F679 of TSC1 sandwich the loop 61-65 of WIPI3 circled in red, reinforcing WIPI3 binding. **(C)** A close-up view of R1743 of TSC2 forming a polar interaction network with residues including R1201, N1205, L1534 and I1537. **(D)** A close-up view of polar interactions of R1200 with T1068 and D1535. **(E)** A close-up view of polar interactions of R611 with surrounding residues including Y602, A607 and E700. **(F)** A close-up view of R905 forming a polar interaction network with C900, S1036, N1037 and Y1033 to stabilise local secondary structure.

TBC1D7 is the third stoichiometric component of the TSCC in all tissues, however its contributions to TSCC activity remain somewhat obscure. It interacts with the TSC1 coiled-coil dimer: TBC1D7 helices 4 and 6 are involved in binding to two TSC1 coiled-coil regions (Figure 1C) (Ramlaul et al., 2021; Yang et al., 2021; Bayly-Jones et al., 2024), and 2 residues V88 and L114 are critical to binding TSC1 (Figure 3A) (Santiago Lima et al., 2014; Nakashima et al., 2007). Three arginine residues, R96, R110, and R121 located at the TBC1D7:TSC1 interface are a mutation hotspot on TBC1D7 (Bayly-Jones et al., 2024; Stenson et al., 2017; Tate et al., 2019). Interestingly, the most frequent missense mutation in TBC1D7 is a surface exposed arginine R167 distant from the TSC1 interface (Tate et al., 2019), however there is no clear functional implication for this particular mutant in connection to TSC (Bayly-Jones et al., 2024; Stenson et al., 2017; Tate et al., 2019). Cell based *TBC1D7* depletion studies have shown modest growth-factor-independent activation of mTORC1, though substantially less than that observed due to loss of *TSC1* or *TSC2*, and this has been attributed to a partial destabilisation of the TSCC leading to reduced GAP activity towards RHEB (Dibble et al., 2012). Reduced TSCC activity observed in the *TBC1D7* knock-out study suggests a non-essential role for maintaining TSCC structural integrity (Schrötter et al., 2022). This might explain why loss-of-function mutants of *TBC1D7* have not been reported in TSC patients (i.e., *TBC1D7* is not *TSC3*), since it is likely the remaining activity towards RHEB of TBC1D7 impaired TSCC is still sufficient to fulfil its biological function. Earlier studies showed that overexpression of TBC1D7 can lead to enhanced ubiquitination of TSC1 and elevated mTORC1 signalling (Alfaiz et al., 2014). Loss of TBC1D7 also results in an increase in mTORC1 signalling and consequently delays the induction of autophagy and enhancement of cell growth under stringent growth conditions (Dibble et al., 2012; González and Hall, 2017). Homozygous loss of *TBC1D7* causes intellectual disability and autosomal-recessive megaencephaly (Alfaiz et al., 2014; Capo-Chichi et al., 2013). Lack of *Tbc1d7* can make brain tissue and neurons in particular more susceptible to overgrowth, leading to abnormal thickness of the cerebral cortex as well as raised neuron-intrinsic mTORC1 signalling, which has also been observed in mouse models with brain-specific *Tsc1* or *Tsc2* knock-outs (Mietzsch et al., 2013; Carso et al., 2012; Crowell et al., 2015). These findings highlight the critical nature of full TSCC function in the regulation of brain development and imply a possible role for TBC1D7 acting as an extra layer in modulating TSC activity.

Most TBC domain–containing proteins function as Rab GTPase–activating proteins (RabGAPs), however TBC1D7 is missing a key helix in the Rab GTPase binding groove and does not have the arginine/glutamine dual-finger residues that are essential for RabGAP activity (Madigan et al., 2018). TBC1D7 itself can be phosphorylated by AKT on serine residue 124. S124 phosphorylation enables 14-3-3 binding, hence stabilising TBC1D7. Furthermore, the sequence immediately upstream of S124 aligns with the canonical β-TrCP degron recognised by the E3 ubiquitin ligase β-TrCP2. Ubiquitination of TBC1D7 leads to protein degradation with reduced steady-state levels observed. AKT activity determines the phosphorylation status of TBC1D7 at the phospho-switch S124, which governs binding to either 14-3-3 or β-TrCP2, and thereby regulating TBC1D7 stability (Madigan et al., 2018).

Finally, a study in hepatocellular carcinoma has suggested that TBC1D7 interacts with kinesin family member 2C (KIF2C), which is highly expressed in some human tumours and a direct target of the Wnt/β-catenin pathway. Interactions of TBC1D7 and KIF2C were reported and linked to possible disruption of the formation of the TSCC, thereby upregulating mTORC1 signalling. Such findings suggest a potential role for KIF2C involved crosstalk between Wnt/β-catenin and mTORC1 signalling (Wei et al., 2021b). More research is required to discover and confirm such novel cellular roles played by TBC1D7 and its binding partners.

## TSC1 – more than merely a stabilising scaffold for the TSCC

TSC1 is necessary for TSCC activity. Whereas it is clear that the catalytic activity of the GAP domain within the TSC2 subunit would be essential for RHEB-GTP hydrolysis, it is less immediately obvious what role TSC1 might play. TSC1 has key functions within the TSCC, including stabilising bound TSC2 and anchoring the TSCC to the periphery of lysosomes and late endosomes. It is also believed that TSC1 moonlights outside of the TSCC, playing non-TSCC roles which require further investigation (Menon et al., 2014; Benvenuto et al., 2000).

TSC1 is essential for the stability of the TSCC. TSC2 becomes heavily ubiquitinated and unstable in the absence of TSC1, which leads to proteasome-mediated degradation. TSC2 has been shown to interact with HERC1, a HECT domain containing ubiquitin E3 ligase. HERC1 contains two regulator of chromosome condensation (RCC)-like domains (RLDs), multiple WD40 repeats, and an E3 ligase HECT domain. It is widely expressed in many tissues and crucial for normal muscle function and neurotransmitter release at the neuromuscular junction (Chong-Kopera et al., 2006; Bachiller et al., 2015). HECT1 utilises its C-terminal domain to interact with the N-terminal region of TSC2, crucial for maintaining the TSC1 interaction. The interaction of TSC1 with TSC2 appears to exclude TSC2 from interacting with HERC1, therefore stabilising the TSCC and preventing TSC2 ubiquitination (Benvenuto et al., 2000; Chong-Kopera et al., 2006). Additionally, TSC1 can be K63 ubiquitin conjugated to its lysine 30 residue by E3 ubiquitin ligase Peli1; the function of TSC1 in suppressing TSC2 ubiquitination is enhanced after Peli1 mediated K63 ubiquitination, which further prevents TSC2 degradation. Conversely, the TSC1 K30A mutant has been shown to fail to inhibit mTORC1 activation or protect TSC2 degradation in FBS-stimulated cells, consistent with the observation that the TSC1 K30A mutant failed to inhibit TSC2 ubiquitination in the presence of Peli1 (Ko et al., 2021). Peli1-mediated TSC1 ubiquitination may expedite the binding of TSC1 to TSC2, thereby stabilising TSC2 and regulating mTORC1.

More recently, a proteomic study implied that TSC1 may play a central role in the formation of the TSCC. The coiled-coil containing region of TSC1 (residues 725–1,047) has been suggested to interact with the phospho-binding pocket of the PIH domain in PIH1D1. PIH1D1 is part of the R2TP chaperone composed of the RUVBL1/RUVBL2 AAA+ ATPases and another adapter protein RPAP3. It functions as co-chaperone of Hsp90 in the assembly of macromolecular complexes (Lynham and Houry, 2022). TSC2 is also reported to make independent interactions with the TPR domains of RPAP3 directly or via Hsp90 and in contact with PIH1D1 (Abéza et al., 2024). Conversely, inactivation of PIH1D1 or the RUVBL1/2 ATPase activity disrupts the association of TSC1 with TSC2. Together these data have implied a model in which the R2TP recruits both TSC1 via PIH1D1 and TSC2 via RPAP3 with the aid of Hsp90, and the chaperone-like activities of RUVBL1/2 are employed presumably to stimulate their assembly. Further investigations are required to validate these results (Abéza et al., 2024).

## TSC1 regulates the localisation of the TSCC

TSC1 is also essential to facilitate relocation of the TSCC to the correct intracellular sites, which is important for the principal regulatory mechanism of TSCC activity through the mTORC1 signalling pathway (Cai et al., 2006; Lee et al., 2007; Zhang et al., 2013). TSC1 functions as the principal mediator of TSCC recruitment to the lysosomal membrane. Recent discoveries have established strong evidence showing that TSC1 has affinity for phosphorylated lipid head groups within the lysosomal membrane. A groundbreaking study by Fitzian and colleagues established that the N-terminal domain of TSC1 has affinity toward phosphatidylinositol phosphate (PIP) lipids (Fitzian et al., 2021). To elucidate the role of the TSC1 PIP binding in TSCC localisation, a PIP binding-deficient mutant was investigated and showed significantly less support for lysosomal TSCC recruitment, and defective rescue of mTORC1 hyperactivity, demonstrating the importance of TSC1 PIP binding for mTORC1 inactivation. Full TSC1-PIP interactions are therefore vital for TSCC function toward mTORC1 inactivation (Fitzian et al., 2021). While TSC1 has been proven capable of binding a wide variety of different PIPs, a recent study of the N-terminal domain of TSC1 by Bayly-Jones and colleagues has revealed more details of the PIP-binding pocket in the TSC1 N-terminal dimer (Figure 1D), and has provided further strong evidence to imply that TSC1 exhibits a specific binding preference for singularly phosphorylated PI3P (Bayly-Jones et al., 2024).

In addition to PIP binding anchoring the TSCC at lysosomes, WIPI3 binds to the very tip of the complex through a conserved motif in TSC1 (Figure 1D) (Bakula et al., 2018). The hydrophobic TSC1 pocket containing residues 659–680 contacts the WIPI3 surface with 2 residues, W676 and F679, orchestrating hydrophobic interactions that clamp to WIPI3’s strand-β5 (Figure 3B), while an electrostatic interaction can be inferred between H678 of TSC1 and D81 of WIPI3. Mutation of TSC1 interacting residues W676, H678, or F679 completely abolishes WIPI3 binding. It has been proposed that the PIP-binding pocket, as well as WIPI3s binding site, together provide the major membrane alignment points for TSCC lysosomal recruitment (Bayly-Jones et al., 2024).

Furthermore, the presence of WIPI3 within the TSCC enables its interaction with FK506-binding protein 51 (FKBP51) (Häusl et al., 2022). FKBP51 is a major modulator of the stress response coordinating diverse pathways to mediate homeostatic control (Taipale et al., 2014). WIPI protein family members WIPI3 and WIPI4 are essential scaffolders of the LKB1/AMPK/TSC signalling network, connecting autophagy and mTOR signalling. For autophagy signalling, FKBP51 recruits LKB1 to the WIPI4-AMPK regulatory platform to induce T172 phosphorylation of AMPK, subsequently triggering autophagy initiation by direct phosphorylation of ULK1 at S555 (Kim et al., 2011). For mTORC1 signalling, FKBP51 is expected to associate with the TSCC/WIPI3 heterocomplex to regulate mTORC1 signalling and thus position FKBP51 as a regulatory switch between autophagy initiation and mTORC1 signalling in response to metabolic challenges (Häusl et al., 2022).

## TSC2-independent functions of TSC1

Apart from its well characterised role in regulating mTORC1 function, TSC1, independent of TSC2 or mTORC1, has been suggested to positively regulate the association of transforming growth factor (TGF)-β receptor type I (TβR-I) with its substrates Smad2/3, leading to their phosphorylation (Thien et al., 2015). TSC1 has been reported to interact with Smad3 via its N-terminal region but not its C-terminal coiled-coil domain, which is essential for mediating TSC1-TSC2 interactions (Ramlaul et al., 2021; Yang et al., 2021; Bayly-Jones et al., 2024). Therefore, distinct TSC1 domains would facilitate TSC1-Smad and TSC1-TSC2 interactions, respectively. TSC1 co-precipitates with TβR-I in *TSC2* knockdown cells, suggesting its involvement in the TGF-β pathway in the absence of TSC2. It is therefore plausible that TSC1 signalling triggers target gene expression and is involved in the control of TGF-β-induced growth arrest and epithelial-to-mesenchymal transition (EMT) through the TGF-β pathway (Thien et al., 2015), although substantial further work is required to validate these results.

In addition to its role in stabilisation of TSC2 and the formation of the TSCC, TSC1 has been reported to function as a co-chaperone of heat-shock protein 90 and to block its ATPase activity to facilitate client loading for other factors (Woodford et al., 2017). The TSC1 homodimer utilises its C-terminal dimerisation region (residues 998–1,164) binding to both protomers of the Hsp90 middle domain. The C-terminus of Hsp90 carries the MEEVD motif which is a highly conserved tetratricopeptide repeat (TPR) domain-binding site mediating interaction with many co-chaperones; no interaction was observed between Hsp90 and TSC1 if the MEEVD motif was removed. Yet the details of MEEVD motif involvement in TSC1 binding remain elusive. TSC1 binds both subunits of the Hsp90 dimer and prevents the activating Hsp90 co-chaperone Aha1 from accessing the middle domain of Hsp90. The Aha1 co-chaperone assists conformational modulations essential for Hsp90 ATPase competence. The TSC1 dimer has higher affinity than the competitor Aha1 toward the same middle domain of Hsp90. When Aha1 is phosphorylated on its Y223 residue by c-Abl, the equilibrium can be driven toward Aha1 binding to replace TSC1 and boost Hsp90 ATPase activity. This study has been suggested to represent a broader function for TSC1 as a facilitator of Hsp90-mediated folding, however it is also quite plausible that these HSP90 interactions are part of the TSC2 stabilisation and TSCC formation pathway, which also involves R2TP, that remain incompletely understood (Woodford et al., 2017).

Finally, TSC1 alone, and not the TSCC, has been reported to have a newly identified critical function in controlling cell–cell adhesion, which is essential for tight junction formation within epithelium, independent of its role in TSC2/mTORC1 regulation (Lai et al., 2021). Myosin 6 (Myo6) is required for TSC1 to function in cell adhesion. Myo6 is an unconventional myosin, the only known motor that moves toward the minus ends of actin filaments, binds β-catenin *in vitro*, and is required to maintain perijunctional actin cytoskeleton connections (Sweeney and Houdusse, 2010). By binding to Myo6, TSC1 appears to help anchor the perijunctional actin cytoskeleton to β-catenin and ZO-1 protein for both tight and adherence junctions. Myo6 binds to a TSC1 segment covering residues 302–430 which is also the identified region for TSC2 binding. The possible competition between Myo6 and TSC2 binding further implies that the role of TSC1 in cell adhesion is independent of TSC2. Junctional TSC1 levels in epithelial tissues are markedly reduced in Crohn’s disease or psoriasis patients, together with the tight junction structure impairment, implying that TSC1 deficiency may also be involved in tight junction-related diseases (Lai et al., 2021).

## Disease-associated variant clusters within the TSCC

TSCC activity can be significantly reduced due to disease-associated *TSC* variants, leading to the accumulation of excessive RHEB-GTP concentrations, and increased mTORC1 activation (Henske et al., 2016). The majority of pathogenic *TSC1* and *TSC2* variants are truncating frameshift or premature termination codon, likely resulting in effectively no TSC1 or TSC2 protein expression from the affected allele due to nonsense-mediated mRNA decay (NMD). Only a minor proportion of pathogenic *TSC1* and *TSC2* variants are missense changes (Supplementary Table S2) (Fokkema et al., 2021; Mozaffari et al., 2009; Hoogeveen-Westerveld et al., 2011; Hoogeveen-Westerveld et al., 2012; Hoogeveen-Westerveld et al., 2013). Nevertheless, analysis of missense changes may provide molecular insights towards better understanding and possibly prediction of the pathogenicity of particular variants. Unsurprisingly, pathogenic *TSC2* missense variants have been found to cluster to the TSC2 catalytic GAP domain due to their loss-of-function impact (Hoogeveen-Westerveld et al., 2011; Hoogeveen-Westerveld et al., 2013). These variants have detrimental effects on protein structure: substitutions either eliminate polar interactions that maintain the protein folding, disrupt the hydrophobic packing of the domain, or are incompatible with the formation of secondary structural elements (Supplementary Table S3) (Hansmann et al., 2020). R1743, P1675, and R1200, located close to the GAP region of TSC2, are amongst the most frequently reported pathogenic variants observed in TSC patients (Fokkema et al., 2021; Stenson et al., 2017; Tate et al., 2019). R1743 forms a polar interaction network with R1201 and N1205 of the neighbouring strand and L1534 and I1537 of the neighbouring helix (Figure 3C). The common pathogenic R1743Q substitution, that replaces the positively-charged arginine with the shorter uncharged glutamine residue, would be expected to prevent this contact network from forming. Similarly, R1200 orchestrates a polar interaction network stabilising the neighbouring strand (T1068) and helix regions (D1535) (Figure 3D). The introduction of the bulky tryptophan side chain due to the common pathogenic R1200W substitution is predicted to sterically disrupt the folding of this entire region. Amino acid substitutions affecting P1675 may disrupt the local secondary structure, interfering with the interaction with TSC1 due to proximity to the TSC1/TSC2 interface. Other than these GAP domain residues, R611 and R905 are particularly frequently mutated residues. R611 sits in the heat repeat region of TSC2, interacting with the side chains of E700 and main chain oxygens of Y602 and A607 to form a network stabilising the local secondary structure (Figure 3E). Mutations of R611 have been reported to disrupt TSCC formation and interfere with TSC2 phosphorylation, impairing mTORC1 signalling (Sweeney and Houdusse, 2010; Nellist et al., 2005a). Substitution with glutamine, would be very likely to impair the interaction with E700. R905 plays a critical role in stabilising local helix conformation and is in a region contacting the neighbouring TSC2 counterpart (Figure 3F). Alterations to R905 do not prevent TSC1-TSC2 binding, but disrupt TSCC RHEB GAP activity and may lead to perturbation of the dimerisation interface presumably impacting TSCC conformation (Nellist et al., 2005b). Relatively fewer disease-related missense variants have been reported in *TSC1* (Supplementary Table S4). Several frequently affected residues are located in the N-terminal region that contains a lipid binding domain which is responsible for proper tethering of the TSCC to the lysosomal surface, where it regulates RHEB (Fitzian et al., 2021). Of those N-terminal *TSC1* variants, K30 is reported to be a crucial K63 ubiquitinated site to further stabilise TSC2 after ubiquitination (Ko et al., 2021). Although mutation of this residue seems to have no impact on the formation of the TSCC structure, it may block essential cellular modifications which disrupt the fine balance of the mTORC1 signalling cascade and lead to the onset of disease (Ko et al., 2021). The coiled-coil regions of individual TSC1s are intertwined to form a dimer and interact with multiple domains of TSC2 (Ramlaul et al., 2021; Yang et al., 2021; Bayly-Jones et al., 2024). Several disease-related *TSC1* missense variants have been reported in the segment containing residues 266–596, however this region cannot be resolved in the currently available structures, implying flexibility, and its role therefore remains opaque. In addition, TSC1 is the binding partner of TBC1D7; two residues, K952 and I954, of TSC1 are essential for maintaining TBC1D7 interactions (Santiago Lima et al., 2014). Therefore, mutations in these regions might potentially lose TBC1D7 binding, leading to deregulated expression patterns in the mTORC1 cascade (Figure 3E).

Several *TSC2* VUS have been reported that are located in the GAP domain. Many of these variants were identified in patients suspected of TSC or having TSC-associated symptoms but showing comparably normal mTORC1 activity in cell-based assays (Hansmann et al., 2020). Such variants might only partially impair TSCC function, and therefore their effects may have proven difficult to detect using *in vitro* functional assays. Although the resulting effects would therefore be expected to be mild, it is likely a small decline in TSCC activity might be sufficient to cause some clinical manifestations. Because of the mild effect of these variants, additional genetic or environmental factors might contribute to clinical penetrance, and it may be expected that other mutations triggering only weak loss of activity of the TSCC will be found to be associated with probable but not readily clinically diagnosable TSC (Hansmann et al., 2020).

## mTORC1 inhibition as a treatment option for TSC

The understanding of TSC’s molecular mechanism led directly to the development of targeted therapies for TSC aimed at inhibiting the mTORC1 pathway. Clinicians soon focused on the already available mTORC1 inhibitor rapamycin (also referred to as sirolimus) in TSC patients (Bissler et al., 2008). *TSC* mutations result in mTORC1 activation, and rapamycin blocks mTORC1 signalling. Rapamycin is produced by the bacterium *Streptomyces hygroscopicus*. It is a clinically approved mTORC1 inhibitor which was capable of being easily repurposed for TSC patients. Rapamycin allosterically inhibits mTORC1 by forming a drug-protein complex with FK506-binding protein 12 (FKBP12). The rapamycin-FKBP12 complex interacts with mTOR on the FKBP12: rapamycin binding (FRB) domain, which inhibits the ability of mTORC1 to phosphorylate downstream substrates (Van Duyne et al., 1993). While rapamycin binds mTORC1 directly, it does not bind the mTORC2 complex as its binding site is obscured by a mTORC2 specific cofactor. The first successful clinical trial of mTORC1 inhibitors was conducted in TSC patients and focused on reducing the size of SEGAs (Krueger et al., 2010). Several rapamycin analogues were developed to enhance the pharmacokinetic profile of their parent compound via a similar mechanism. Everolimus has superior pharmacokinetics and more robust clinical trial experience in oncology and TSC than rapamycin (MacKeigan and Krueger, 2015). Temsirolimus is a prodrug of rapamycin and it has been approved for intravenous treatment for advanced renal cell carcinoma (Hudes et al., 2007). Ridaforolimus (also known as deforolimus) is an mTORC1 inhibitor under investigation, which shows antitumour activity against metastatic sarcoma (Mita et al., 2008). Rapamycin and synthetic mTOR-inhibiting compounds known as rapalogues, such as everolimus, temsirolimus and ridaforolimus are classed as first generation mTOR inhibitors. mTORC1 inhibitors are useful treatments for some of the tumours associated with TSC including sirolimus for pulmonary LAM and renal AML (Franz et al., 2013), and everolimus for AML and inoperable SEGAs (Franz et al., 2013; Bissler et al., 2013). Both drugs can reduce tumour burden and provide symptomatic relief for patients. However, mTORC1 inhibitors display reduced efficacy in neurological disorders due to their limited penetration across the intact blood–brain barrier (Crino, 2016). mTORC1 inhibitors have low level toxicity, and can cause mouth ulceration and stomatitis, with additional risks due to immunosuppression, rapamycin came to widespread use as a strong immunosuppressant due to its ability to prevent leukocyte growth, and thus the possibility of severe infection (Previtali et al., 2023). All patients reported at least one adverse event in 5 years during the everolimus treatment trial. Common side effects included upper respiratory infections (86%), aphthous oral ulcers (mucositis) and stomatitis (86%), sinusitis (46%), and otitis media (36%). None of the events were severe enough to disrupt everolimus treatment and the incidence of adverse effects diminished with longer everolimus exposure (Franz et al., 2015). Another issue with the use of rapalogues is that they are cytostatic agents, since they are targeting mTORC1 to ease the cell growth but not repairing/reactivating TSC genes. Lesion regrowth is therefore expected on the cessation of therapy (Bissler et al., 2008; Tran and Zupanc, 2015). Protracted interruption of mTORC1 inhibitor treatment in TSC leads to relapse of tumours or seizures. Consequently, long-term treatment is required for TSC-related manifestations (Franz et al., 2016; Mingarelli et al., 2018).

Since rapalogues have a limited ability to fully regulate mTORC1 activity, research has gone into developing more potent compounds that can block the catalytic activity of mTORC1. ATP-competitive inhibitors as the second generation of mTOR inhibitors have been considered ideal candidates for this purpose. ATP-competitive inhibitors interact with the ATP-binding pocket of the mTOR kinase domain, preventing the docking of ATP required for the phosphorylation process. While ATP-competitive inhibitors are more potent in repressing mTORC1, they may be impractical when considered as a long-term therapy for TSC in addition to possessing a greater risk of toxicity, since the total blockage is extremely harmful to healthy tissues and leads to adverse effects. mTORC2, the rapamycin-insensitive mTOR protein kinase complex involved in the regulation of the actin cytoskeleton, and cell survival signalling, acts as the secondary kinase in AKT regulation and is also blocked by these second-generation inhibitors (Apsel et al., 2008; Ali et al., 2022).

ATP-competitive mTOR inhibitors have yet to reach their therapeutic potential and trials remain ongoing (Basu et al., 2015). The third-generation mTOR inhibitor RapaLink exploits the juxtaposition of two drug-binding pockets to create a bivalent interaction with the target kinase (Rodrik-Outmezguine et al., 2016). This hybrid molecule contains both rapamycin and an mTOR ATP-competitive kinase inhibitor, which are linked via a non-perturbing, strain-free crosslinker of optimal length. The linker permits the chemical to connect with the FRB domain of mTOR through the interaction with FKBP12, as well as to reach the kinase domain of mTOR, allowing it to serve as an ATP-competitive inhibitor. By combining this with a mTORC1 selective ATP-competitive inhibitor, it has proven possible to create bi-steric versions with several-fold enhanced selectivity for mTORC1 over mTORC2. These third-generation mTOR inhibitors, including RMC-6272 and its clinical counterpart RMC-5552, show robust anti-tumour activity either alone or when combined with other treatments in several preclinical cancer models (Bhattacharyya et al., 2024; Burnett et al., 2023; Lee et al., 2021). RMC-5552 has over 30-fold selectivity for mTORC1 over mTORC2 and is currently under clinical evaluation (trials NCT04774952 and NCT05557292). Polysome profiling of TSC patient-derived isogenic neural progenitor cells (NPC) revealed numerous changes in mRNA levels and translation associated with *TSC1*-loss. Treatment of NPCs using rapamycin partially rescues *TSC1*-associated translation, yet most genes related to neural activity and synaptic regulation remained unchanged. Strikingly, RMC-6272 can rescue early neurodevelopmental phenotypes such as proliferation and neurite outgrowth in *TSC1* ^−/−^ NPCs which were previously rapamycin-insensitive. RapaLink could therefore be a promising treatment strategy for neural symptoms of TSC via the mTORC1 axis (Aksoylu et al., 2023).

## New approaches to treat TSC

Instead of inhibiting mTORC1, an alternative strategy currently under evaluation for TSC is to exploit the metabolic vulnerabilities of mTORC1 hyperactive cells, which can be leveraged to yield targeted cytotoxicity (Schonthal, 2012; Johnson et al., 2015; McEneaney and Tee, 2019). mTORC1 hyperactive cells have enhanced basal endoplasmic reticulum (ER) stress, which is due to elevated levels of mTORC1-directed protein synthesis that places a burden on the protein folding capacity of the ER. The unfolded protein response (UPR) will be activated due to ER stress, which aims to downregulate protein synthesis and restore protein folding, therefore maintaining cellular homeostasis. Alternatively, cell death is initiated following excessive ER stress over a prolonged period of time (Schönthal, 2012). C/EBP homologous protein (CHOP), also known as growth arrest and DNA damage inducible gene 153 (GADD153), is a key player in the ER stress response (Oyadomari and Mori, 2004). CHOP directly activates expression of GADD34, a protein phosphatase 1 (PP1) regulator which causes PP1-mediated eIF2α dephosphorylation, releasing the translational block, and thereby enhancing protein synthesis to promote death-associated mechanisms (Brush et al., 2003). mTORC1 hyperactivation specifically sensitises tumour cells to drug-induced ER stress and can lead to autophagy-independent cell death (Brush et al., 2003). TSCC-deficient cells have previously been shown to be compromised in their ER stress response, since mTORC1 further enhances the burden of ER stress through autophagy repression, as autophagy is employed by the cell for unfolded protein removal to restore ER homeostasis (Kang et al., 2011). This is consistent with the finding that TSC patient-derived AML cells have elevated levels of ER stress and that this can be exacerbated further by a proteasome inhibitor MG-132 (Siroky et al., 2012). Recent studies showed that combinatorial therapy with an ER stress inducer, nelfinavir, in the presence of an autophagy inhibitor, chloroquine (Johnson et al., 2015), or a proteasomal inhibitor, bortezomib (Johnson et al., 2018), showed promise in both *in vitro* and *in vivo* models of TSC to reduce tumour size and to selectively kill TSC-deficient cells. Nelfinavir and bortezomib were effective at killing the tumours *in vitro*, and cell recovery was not observed following removal of the drug combination (Johnson et al., 2018). These studies demonstrate a potent cytotoxic response to TSC-diseased cells with ER stress inducers, which are tolerated by the normal cells with an intact TSCC signalling pathway, however, more evidence of efficacy is required, particularly toward TSC, before this avenue can be pursued as a possible TSC therapy.

Rapalogues are usually reasonably well tolerated, however lifelong therapy causes immune suppression and potentially compromises early brain development including axon guidance, neuronal growth, synapse formation, and myelination (Chapman and Chi, 2014; Wang et al., 2017; Jeong and Wong, 2016). There is a clear need to develop other therapeutic approaches for TSC beyond mTORC1 inhibition. Proof of concept gene therapy for TSC has been evaluated in mouse models of TSC using an adeno-associated virus (AAV) vector carrying the complement for either a full-length *TSC1* or a functional engineered human *TSC2*, respectively (Prabhakar et al., 2019; Cheah et al., 2021). By intravascular injection of AAV9 vectors carrying the required TSC proteins into the corresponding stochastic *Tsc1*-floxed or *Tsc2*-floxed mouse models on day 21, most experimental mice significantly extended lifespans, the size of ependymal and subependymal lesions observed in their brains was reduced and cell proliferation in the sub-ependymal zone became normalised with a clear reduction of mTORC1 driven S6 kinase phosphorylation (Prabhakar et al., 2019; Cheah et al., 2021). The substantial advantage of AAV or similar gene therapies would be the potential for a single vector injection yielding long-term transgene expression in non-dividing cells. It is hoped that gene replacement therapy might minimise the use of more problematic standard-of-care procedures in young children and provide a single administration with long-lasting benefits. Certain serotypes of AAV including AAV9 and AAVrh8, can cross the blood-brain barrier as well as deliver to peripheral tissues (Yang et al., 2014). Therefore, the replacement gene could be delivered to multiple tissues, including brain, kidney, liver, and lungs via intravascular administration, which might diminish the likelihood that somatic mutations in TSC genes later in life would lead to disruptive hamartomas.

## iPSCs and CRISPR editing: towards personalised therapies for TSC

Recent advances in stem cell biology with the derivation of human-induced pluripotent stem cells (iPSC) from the somatic cells of patients have also opened new avenues into the study of TSC (Nadadhur et al., 2019; Li et al., 2017; Guo et al., 2025). This approach combined with gene-editing tools such as CRISPR/Cas9 offers the advantage of preserving patient-specific genetic background and the ability to create isogenic controls by gene correction (Guo et al., 2025). The patient cell line and the isogenic control can be differentiated into the cell type of interest to model various aspects of TSC, since TSC can affect multiple organ systems and leads to different manifestations. By combining iPSC and CRISPR-based genome editing, a human cerebral organoid model for tuberous sclerosis complex has been generated and led to the identification of a specific neural stem cell type, caudal late interneuron progenitor (CLIP) cells, responsible for initiating both tumour and cortical tuber formation (Eichmüller et al., 2022). Instead of a second somatic mutation of *TSC2* during tumour progression, CLIP cells lose their healthy *TSC2* allele due to copy-neutral LOH, the exchange of large genomic regions between homologous chromosomes. Furthermore, CLIP cells require epidermal growth factor receptor signalling to proliferate, and inhibition of EGFR can revert the TSC phenotype, suggesting a potential pathway for therapeutic intervention. The technology could serve as a valuable tool for disease modelling and might facilitate further testing of therapeutics and the identification of opportunities for new treatments such as novel gene therapy approaches to cure TSC.

## Continuing the fight against TSC

The TSCC is a key regulator of cell growth and metabolism, and its dysfunction lies at the heart of TSC pathogenesis. In recent years, significant breakthroughs have been made in the study of the canonical TSC signalling pathway at a molecular level. The resolution of the structure of the TSCC has been a key breakthrough that now allows mutational data to be interpreted with reference to a protein model, and, with future improvements upon the current structure, these insights will over time come to underpin all molecular understanding of TSC pathogenesis. A breakthrough of similar magnitude has been the understanding that TSCC recruitment to the lysosome is based to a large extent on phospholipid recognition, providing a new and important role for TSC1 and directly involving the WIPI apparatus in TSC signalling. However, great strides in our molecular understanding must still be taken. The key remaining part of the puzzle for the canonical TSC signalling pathway from the molecular perspective remains the connection of the TSCC’s post-translational modification to its newly-understood localisation apparatus. It is well established that these modifications act as a switch to direct the TSCC between the lysosome and the cytoplasm, but the molecular details of this coupling remain elusive: hopefully this will bring us to a complete description of the canonical pathway.

Numerous observations of non-canonical TSC signalling activities have also cropped up in recent years. Certain of these, such as the chaperone related activities of TSC1 and TSC2, may be explained by homeostasis of the canonical signalling pathway, others, such as the independent activity of TSC1 at tight junctions, may represent similar mechanisms to the canonical pathway occurring in a different context, while others still, such as the reproducible involvement of the TSCC in stress-granule formation, may represent cross-talk with previously unconsidered pathways such as autophagy. What is clear throughout these traces is that there is much yet to be discovered beyond the canonical pathway of TSC signalling that provides essential future avenues for the field.

Finally, despite recent advances in the mTOR inhibitors improving the quality of life for individuals with TSC, challenges remain in translating the many new molecular insights being made into durable clinical benefits. A large part of this is because such pathogenic variants leading to loss of functional protein are almost impossible to patch up within the cell, necessitating the targeting of downstream mTORC1 signalling instead, an approach guaranteed to have side-effects. While the eventual treatment of TSC as a condition seems likely to await advances in either gene therapy to replace the activity of the defective TSCC, or targeted killing of TSCC deficient cells to prevent the growth of benign tumours, continued research into the intricacies of TSCC signalling, as well as novel therapeutic approaches, remains critical to improve outcomes in the near term, through better understanding of pathogenesis, for individuals affected by this multifaceted disorder.

## References

[B1] Abéza C. Busse P. Paiva A. C. F. Chagot M. E. Schneider J. Robert M. C. (2024). The HSP90/R2TP quaternary chaperone scaffolds assembly of the TSC complex. J. Mol. Biol. 436 (23), 168840. 10.1016/j.jmb.2024.168840 39490680

[B2] Aksoylu I. S. Martin P. Robert F. Szkop K. J. Redmond N. E. Bhattacharyya S. (2023). Translatome analysis of Tuberous sclerosis complex 1 patient-derived neural progenitor cells reveals rapamycin-dependent and independent alterations. Mol. Autism 14, 39. 10.1186/s13229-023-00572-3 37880800 PMC10601155

[B3] Alfaiz A. Micale L. Mandriani B. Augello B. Pellico M. T. Chrast J. (2014). TBC1D7 mutations are associated with intellectual disability, macrocrania, patellar dislocation, and celiac disease. Hum. Mutat. 35 (35), 447–451. 10.1002/humu.22529 24515783

[B4] Ali E. S. Mitra K. Akter S. Ramproshad S. Mondal B. Khan I. N. (2022). Recent advances and limitations of mTOR inhibitors in the treatment of cancer. Cancer Cell. Int. 22, 284. 10.1186/s12935-022-02706-8 36109789 PMC9476305

[B5] Amemiya Y. Ioi Y. Araki M. Kontani K. Maki M. Shibata H. (2024). Calmodulin enhances mTORC1 signaling by preventing TSC2-Rheb binding. J. Biol. Chem. 21, 108122. 10.1016/j.jbc.2024.108122 PMC11787510 39716490

[B6] Apsel B. Blair J. A. Gonzalez B. Nazif T. M. Feldman M. E. Aizenstein B. (2008). Targeted polypharmacology: discovery of dual inhibitors of tyrosine and phosphoinositide kinases. Nat. Chem. Biol. 4, 691–699. 10.1038/nchembio.117 18849971 PMC2880455

[B7] Arbones M. L. Thomazeau A. Nakano-Kobayashi A. Hagiwara M. Delabar J. M. (2019). DYRK1A and cognition: a lifelong relationship. Pharmacol. Ther. 194, 199–221. 10.1016/j.pharmthera.2018.09.010 30268771

[B8] Bachiller S. Rybkina T. Porras-García E. Pérez-Villegas E. Tabares L. Armengol J. A. (2015). The HERC1 E3 ubiquitin ligase is essential for normal development and for neurotransmission at the mouse neuromuscular junction. Cell. Mol. Life Sci. 72 (15), 2961–2971. 10.1007/s00018-015-1878-2 25746226 PMC11113414

[B9] Bakula D. Mueller A. J. Proikas-Cezanne T. (2018). WIPI β-propellers function as scaffolds for STK11/LKB1-AMPK and AMPK-Related kinase signaling in autophagy. Autophagy 14 (6), 1082–1083. 10.1080/15548627.2017.1382784 28976799 PMC6103416

[B10] Ballif B. A. Roux P. P. Gerber S. A. MacKeigan J. P. Blenis J. Gygi S. P. (2005). Quantitative phosphorylation profiling of the ERK/p90 ribosomal S6 kinase-signaling cassette and its targets, the tuberous sclerosis tumor suppressors. Proc. Natl. Acad. Sci. U. S. A. 102, 667–672. 10.1073/pnas.0409143102 15647351 PMC545566

[B11] Balzer F. Menetrier P. (1885). Etude sur un cas d’adénomes sébacés de la face et du cuir chevelu. Arch. Physiol. Norm. Pathol. 6, 564–576.

[B12] Basu B. Dean E. Puglisi M. Greystoke A. Ong M. Burke W. (2015). First-in-human pharmacokinetic and pharmacodynamic study of the dual m-TORC 1/2 inhibitor AZD2014. Clin. Cancer Res. 21, 3412–3419. 10.1158/1078-0432.CCR-14-2422 25805799 PMC4512239

[B13] Bayly-Jones C. Lupton C. J. D’Andrea L. Chang Y. G. Jones G. D. Steele J. R. (2024). Structure of the human TSC:WIPI3 lysosomal recruitment complex. Sci. Adv. 10 (47), eadr5807. 10.1126/sciadv.adr5807 39565846 PMC11578170

[B14] Benvenuto G. Li S. Brown S. J. Braverman R. Vass W. C. Cheadle J. P. (2000). The tuberous sclerosis-1 (TSC1) gene product hamartin suppresses cell growth and augments the expression of the TSC2 product tuberin by inhibiting its ubiquitination. Oncogene 19, 6306–6316. 10.1038/sj.onc.1204009 11175345

[B15] Bernstein J. (1991). Renal involvement in tuberous sclerosis. Ann. N. Y. Acad. Sci. 615, 36–49. 10.1111/j.1749-6632.1991.tb37746.x 2039157

[B16] Bhattacharyya S. Oblinger J. L. Beauchamp R. L. Kosa L. Robert F. Plotkin S. R. (2024). Preclinical evaluation of the third-generation, bi-steric mechanistic target of rapamycin complex 1-selective inhibitor RMC-6272 in NF2-deficient models. Neurooncol Adv. 6 (1), vdae024. 10.1093/noajnl/vdae024 38476930 PMC10929445

[B17] Bissler J. J. Kingswood J. C. (2004). Renal angiomyolipomata. Kidney Int. 66, 924–934. 10.1111/j.1523-1755.2004.00838.x 15327383

[B18] Bissler J. J. Kingswood J. C. Radzikowska E. Zonnenberg B. A. Frost M. Belousova E. (2013). Everolimus for angiomyolipoma associated with Tuberous sclerosis complex or sporadic lymphangioleiomyomatosis (EXIST-2): a multicentre, randomised, double-blind, placebo-controlled trial. Lancet 381 (9869), 817–824. 10.1016/S0140-6736(12)61767-X 23312829

[B19] Bissler J. J. McCormack F. X. Young L. R. Elwing J. M. Chuck G. Leonard J. M. (2008). Sirolimus for angiomyolipoma in Tuberous sclerosis complex or lymphangioleiomyomatosis. N. Engl. J. Med. 358 (2), 140–151. 10.1056/NEJMoa063564 18184959 PMC3398441

[B20] Blanc R. S. Richard S. (2017). Arginine methylation: the coming of age. Mol. Cell. 65, 8–24. 10.1016/j.molcel.2016.11.003 28061334

[B21] Bourneville D. M. (1880). Sclerose tubereuse des circonvolutions cerebrales: idiotie et epilepaie hemiplegique. Arch. Neural. (Paris) (1), 81–91.

[B22] Bourneville D. M. Brissaud E. (1881). Encephalite ou sclerose tubereuse des circonvolutions cerebrales. Arch. Neural. (1), 390–410.

[B23] Brigo F. Lattanzi S. Trinka E. Nardone R. Bragazzi N. L. Ruggieri M. (2018). First descriptions of tuberous sclerosis by désiré-magloire bourneville (1840-1909). Neuropathology 38 (6), 577–582. 10.1111/neup.12515 30215888

[B24] Brush M. H. Weiser D. C. Shenolikar S. (2003). Growth arrest and DNA damage-inducible protein GADD34 targets protein phosphatase 1 alpha to the endoplasmic reticulum and promotes dephosphorylation of the alpha subunit of eukaryotic translation initiation factor 2. Mol. Cell. Biol. 23, 1292–1303. 10.1128/mcb.23.4.1292-1303.2003 12556489 PMC141149

[B25] Burnett G. L. Yang Y. C. Aggen J. B. Pitzen J. Gliedt M. K. Semko C. M. (2023). Discovery of RMC-5552, a selective bi-steric inhibitor of mTORC1, for the treatment of mTORC1-activated tumors. J. Med. Chem. 66 (1), 149–169. 10.1021/acs.jmedchem.2c01658 36533617 PMC9841523

[B26] Cai S. L. Tee A. R. Short J. D. Bergeron J. M. Kim J. Shen J. (2006). Activity of TSC2 is inhibited by AKT-Mediated phosphorylation and membrane partitioning. J. Cell. Biol. 173, 279–289. 10.1083/jcb.200507119 16636147 PMC2063818

[B27] Calixto A. R. Moreira C. Pabis A. Kötting C. Gerwert K. Rudack T. (2019). GTP hydrolysis without an active site base: a unifying mechanism for ras and related GTPases. J. Am. Chem. Soc. 141 (27), 10684–10701. 10.1021/jacs.9b03193 31199130

[B28] Capo-Chichi J.-M. Tcherkezian J. Hamdan F. F. Decarie J. C. Dobrzeniecka S. Patry L. (2013). Disruption of TBC1D7, a subunit of the TSC1-TSC2 protein complex, in intellectual disability and megalencephaly. J. Med. Genet. 50, 740–744. 10.1136/jmedgenet-2013-101680 23687350

[B29] Carson R. P. Van Nielen D. L. Winzenburger P. A. Ess K. C. (2012). Neuronal and glia abnormalities in TSC1-deficient forebrain and partial rescue by rapamycin. Neurobiol. Dis. 45, 369–380. 10.1016/j.nbd.2011.08.024 21907282 PMC3225598

[B30] Chapman N. M. Chi H. (2014). mTOR signaling, tregs and immune modulation. Immunotherapy 6 (12), 1295–1311. 10.2217/imt.14.84 25524385 PMC4291176

[B31] Cheah P. S. Prabhakar S. Yellen D. Beauchamp R. L. Zhang X. Kasamatsu S. (2021). Gene therapy for Tuberous sclerosis complex type 2 in a mouse model by delivery of AAV9 encoding a condensed form of tuberin. Sci. Adv. 7 (2), eabb1703. 10.1126/sci.adv.abb1703 33523984 PMC7793581

[B32] Cheng M. H. Jansen R. P. (2017). A jack of all trades: the RNA-Binding protein vigilin. Wiley Interdiscip. Rev. RNA 8, e1448. 10.1002/wrna.1448 28975734

[B33] Chong-Kopera H. Inoki K. Li Y. Zhu T. Garcia-Gonzalo F. R. Rosa J. L. (2006). TSC1 stabilizes TSC2 by inhibiting the interaction between TSC2 and the HERC1 ubiquitin ligase. J. Biol. Chem. 281 (13), 8313–8316. 10.1074/jbc.C500451200 16464865

[B34] Condon K. J. Sabatini D. M. (2019). Nutrient regulation of mTORC1 at a glance. J. Cell. Sci. 132 (21), jcs222570. 10.1242/jcs.222570 31722960 PMC6857595

[B35] Crino P. B. (2016). The mTOR signalling Cascade: paving new roads to cure neurological disease. Nat. Rev. Neurol. 12 (7), 379–392. 10.1038/nrneurol.2016.81 27340022

[B36] Crowell B. Hwa Lee G. Nikolaeva I. Dal Pozzo V. D’Arcangelo G. (2015). Complex neurological phenotype in mutant mice lacking Tsc2 in excitatory neurons of the developing forebrain. eNeuro 2, ENEURO.0046–15.2015. 10.1523/ENEURO.0046-15.2015 PMC4676199 26693177

[B37] Curatolo P. Bombardieri R. Jozwiak S. (2008). Tuberous sclerosis. Lancet 372 (9639), 657–668. 10.1016/S0140-6736(08)61279-9 18722871

[B38] Demetriades C. Doumpas N. Teleman A. A. (2014). Regulation of TORC1 in response to amino acid starvation via lysosomal recruitment of TSC2. Cell. 156 (4), 786–799. 10.1016/j.cell.2014.01.024 24529380 PMC4346203

[B39] Demetriades C. Plescher M. Teleman A. A. (2016). Lysosomal recruitment of TSC2 is a universal response to cellular stress. Nat. Commun. 7, 10662. 10.1038/ncomms10662 26868506 PMC4754342

[B40] de Vries P. J. Whittemore V. H. Leclezio L. Byars A. W. Dunn D. Ess K. C. (2015). Tuberous sclerosis associated neuropsychiatric disorders (TAND) and the TAND checklist. Pediatr. Neurol. 52, 25–35. 10.1016/j.pediatrneurol.2014.10.004 25532776 PMC4427347

[B41] Dibble C. C. Elis W. Menon S. Qin W. Klekota J. Asara J. M. (2012). TBC1D7 is a third subunit of the TSC1- TSC2 complex upstream of mTORC1. Mol. Cell. 47, 535–546. 10.1016/j.molcel.2012.06.009 22795129 PMC3693578

[B42] Eichmüller O. L. Corsini N. S. Vértesy Á. Morassut I. Scholl T. Gruber V. E. (2022). Amplification of human interneuron progenitors promotes brain tumors and neurological defects. Science 375 (6579), eabf5546. 10.1126/science.abf5546 35084981 PMC7613689

[B43] European Chromosome 16 Tuberous Sclerosis Consortium (1993). Identification and characterization of the tuberous sclerosis gene on chromosome 16. Cell. 75 (7), 1305–1315. 10.1016/0092-8674(93)90618-z 8269512

[B44] Feng R. Liu F. Li R. Zhou Z. Lin Z. Lin S. (2024). The rapid proximity labeling system PhastID identifies ATP6AP1 as an unconventional GEF for rheb. Cell. Res. 34, 355–369. 10.1038/s41422-024-00938-z 38448650 PMC11061317

[B45] Fitzian K. Brückner A. Brohée L. Zech R. Antoni C. Kiontke S. (2021). TSC1 binding to lysosomal PIPs is required for TSC complex translocation and mTORC1 regulation. Mol. Cell. 81 (13), 2705–2721.e8. 10.1016/j.molcel.2021.04.019 33974911

[B46] Fokkema IFAC Kroon M. López Hernández J. A. Asscheman D. Lugtenburg I. Hoogenboom J. (2021). The LOVD3 platform: efficient genome-wide sharing of genetic variants. Eur. J. Hum. Genet. 29, 1796–1803. 10.1038/s41431-021-00959-x 34521998 PMC8632977

[B47] Franz D. N. Agricola K. Mays M. Tudor C. Care M. M. Holland-Bouley K. (2015). Everolimus for subependymal giantcell astrocytoma: 5-Year final analysis. Ann. Neurol. 78, 929–938. 10.1002/ana.24523 26381530 PMC5063160

[B48] Franz D. N. Belousova E. Sparagana S. Bebin E. M. Frost M. Kuperman R. (2013). Efficacy and safety of everolimus for subependymal giant cell astrocytomas associated with Tuberous sclerosis complex (EXIST-1): a multicentre, randomised, placebo-controlled phase 3 trial. Lancet 381 (9861), 125–132. 10.1016/S0140-6736(12)61134-9 23158522

[B49] Franz D. N. Belousova E. Sparagana S. Bebin E. M. Frost M. D. Kuperman R. (2016). Long-term use of everolimus in patients with Tuberous sclerosis complex: final results from the EXIST-1 study. PLoS One 11 (6), e0158476. 10.1371/journal.pone.0158476 27351628 PMC4924870

[B50] Fryer A. E. Chalmers A. Connor J. M. Fraser I. Povey S. Yates A. D. (1987). Evidence that the gene for tuberous sclerosis is on chromosome 9. Lancet 8534, 659–661. 10.1016/s0140-6736(87)90416-8 2882085

[B51] Gen S. Matsumoto Y. Kobayashi K. I. Suzuki T. Inoue J. Yamamoto Y. (2020). Stability of Tuberous sclerosis complex 2 is controlled by methylation at R1457 and R1459. Sci. Rep. 10, 21160. 10.1038/s41598-020-78274-6 33273660 PMC7713242

[B52] Gomez M. R. (1979). Tuberous sclerosis. 1st ed. New York, NY: Raven Press.

[B53] González A. Hall M. N. (2017). Nutrient sensing and TOR signaling in yeast and mammals. EMBO J. 36, 397–408. 10.15252/embj.201696010 28096180 PMC5694944

[B54] Guo G. Moser M. Chifamba L. Julian D. Teierle S. Rajappa P. (2025). CRISPR-Cas9-Mediated correction of TSC2 pathogenic variants in iPSCs from patients with Tuberous sclerosis complex type 2. CRISPR J. 8 (1), 60–70. 10.1089/crispr.2024.0079 39654514

[B55] Hansmann P. Brückner A. Kiontke S. Berkenfeld B. Seebohm G. Brouillard P. (2020). Structure of the TSC2 GAP domain: mechanistic insight into catalysis and pathogenic mutations. Structure 28 (8), 933–942.e4. 10.1016/j.str.2020.05.008 32502382

[B56] Häusl A. S. Bajaj T. Brix L. M. Pöhlmann M. L. Hafner K. De Angelis M. (2022). Mediobasal hypothalamic FKBP51 acts as a molecular switch linking autophagy to whole-body metabolism. Sci. Adv. 8, eabi4797. 10.1126/sciadv.abi4797 35263141 PMC8906734

[B57] He X. Wang Q. X. Wei D. Lin Y. Zhang X. Wu Y. (2025). Lysosomal EGFR acts as a Rheb-GEF independent of its kinase activity to activate mTORC1. Cell. Res. 10.1038/s41422-025-01110-x PMC12205066 40259053

[B58] Henske E. P. Jóźwiak S. Kingswood J. C. Sampson J. R. Thiele E. A. (2016). Tuberous sclerosis complex. Nat. Rev. Dis. Prim. 26 (2), 16035. 10.1038/nrdp.2016.35 27226234

[B59] Henske E. P. McCormack F. X. (2012). Lymphangioleiomyomatosis - a wolf in sheep’s clothing. J. Clin. Invest. 122, 3807–3816. 10.1172/JCI58709 23114603 PMC3484429

[B60] Holmes G. L. Stafstrom C. E. Tuberous Sclerosis Study Group (2007). Tuberous sclerosis complex and epilepsy: recent developments and future challenges. Epilepsia 48, 617–630. 10.1111/j.1528-1167.2007.01035.x 17386056

[B61] Hoogeveen-Westerveld M. Ekong R. Povey S. Karbassi I. Batish S. D. den Dunnen J. T. (2012). Functional assessment of TSC1 missense variants identified in individuals with Tuberous sclerosis complex. Hum. Mutat. 33 (3), 476–479. 10.1002/humu.22007 22161988

[B62] Hoogeveen-Westerveld M. Ekong R. Povey S. Mayer K. Lannoy N. Elmslie F. (2013). Functional assessment of TSC2 variants identified in individuals with Tuberous sclerosis complex. Hum. Mutat. 34 (1), 167–175. 10.1002/humu.22202 22903760

[B63] Hoogeveen-Westerveld M. Wentink M. van den Heuvel D. Mozaffari M. Ekong R. Povey S. (2011). Functional assessment of variants in the TSC1 and TSC2 genes identified in individuals with Tuberous sclerosis complex. Hum. Mutat. 32 (4), 424–435. 10.1002/humu.21451 21309039

[B64] Huang J. Manning B. D. (2008). The TSC1-TSC2 complex: a molecular switchboard controlling cell growth. Biochem. J. 412, 179–190. 10.1042/BJ20080281 18466115 PMC2735030

[B65] Hudes G. Carducci M. Tomczak P. Dutcher J. Figlin R. Kapoor A. (2007). Temsirolimus, interferon alfa, or both for advanced renal-cell carcinoma. N. Engl. J. Med. 356 (22), 2271–2281. 10.1056/NEJMoa066838 17538086

[B66] Inoki K. Li Y. Zhu T. Wu J. Guan K. L. (2002). TSC2 is phosphorylated and inhibited by akt and suppresses mTOR signalling. Nat. Cell. Biol. 4, 648–657. 10.1038/ncb839 12172553

[B67] Inoki K. Ouyang H. Zhu T. Lindvall C. Wang Y. Zhang X. (2006). TSC2 integrates wnt and energy signals via a coordinated phosphorylation by AMPK and GSK3 to regulate cell growth. Cell. 126, 955–968. 10.1016/j.cell.2006.06.055 16959574

[B68] Jeong A. Wong M. (2016). mTOR inhibitors in children: current indications and future directions in neurology. Curr. Neurol. Neurosci. Rep. 16 (12), 102. 10.1007/s11910-016-0708-8 27815691

[B69] Johnson C. E. Dunlop E. A. Seifan S. McCann H. D. Hay T. Parfitt G. J. (2018). Loss of Tuberous sclerosis complex 2 sensitizes tumors to nelfinavir-bortezomib therapy to intensify endoplasmic reticulum stress-induced cell death. Oncogene 37 (45), 5913–5925. 10.1038/s41388-018-0381-2 29980790

[B70] Johnson C. E. Hunt D. K. Wiltshire M. Herbert T. P. Sampson J. R. Errington R. J. (2015). Endoplasmic reticulum stress and cell death in mTORC1-overactive cells is induced by nelfinavir and enhanced by chloroquine. Mol. Oncol. 9 (9), 675–688. 10.1016/j.molonc.2014.11.005 25498902 PMC5528710

[B71] Joinson C. O’Callaghan F. J. Osborne J. P. Martyn C. Harris T. Bolton P. F. (2003). Learning disability and epilepsy in an epidemiological sample of individuals with Tuberous sclerosis complex. Psychol. Med. 33, 335–344. 10.1017/s0033291702007092 12622312

[B72] Kandt R. S. Haines J. L. Smith M. Northrup H. Gardner R. J. M. Short M. P. (1992). Linkage of an important gene locus for tuberous sclerosis to a chromosome 16 marker for polycystic kidney disease. Nat. Genet. 2, 37–41. 10.1038/ng0992-37 1303246

[B73] Kang Y. J. Lu M. K. Guan K. L. (2011). The TSC1 and TSC2 tumor suppressors are required for proper ER stress response and protect cells from ER stress-induced apoptosis. Cell. Death Differ. 18, 133–144. 10.1038/cdd.2010.82 20616807 PMC3131877

[B74] Kedersha N. Anderson P. (2007). Mammalian stress granules and processing bodies. Methods Enzymol. 431, 61–81. 10.1016/S0076-6879(07)31005-7 17923231

[B75] Kim G. E. Kass D. A. (2016). Cardiac phosphodiesterases and their modulation for treating heart disease. Handb. Exp. Pharmacol. 243, 249–269. 10.1007/164_2016_82 PMC5665023 27787716

[B76] Kim J. Guan K. L. (2019). mTOR as a central hub of nutrient signalling and cell growth. Nat. Cell. Biol. 21, 63–71. 10.1038/s41556-018-0205-1 30602761

[B77] Kim J. Kundu M. Viollet B. Guan K. L. (2011). AMPK and mTOR regulate autophagy through direct phosphorylation of Ulk1. Nat. Cell. Biol. 13 (2), 132–141. 10.1038/ncb2152 21258367 PMC3987946

[B78] Knudson A. G. Jr (1971). Mutation and cancer: statistical study of retinoblastoma. Proc. Natl. Acad. Sci. U. S. A. 68, 820–823. 10.1073/pnas.68.4.820 5279523 PMC389051

[B79] Ko C. J. Zhang L. Jie Z. Zhu L. Zhou X. Xie X. (2021). The E3 ubiquitin ligase Peli1 regulates the metabolic actions of mTORC1 to suppress antitumor T cell responses. EMBO J. 40 (2), e104532. 10.15252/embj.2020104532 33215753 PMC7809702

[B80] Kosmas K. Filippakis H. Khabibullin D. Turkiewicz M. Lam H. C. Yu J. (2021). TSC2 interacts with HDLBP/vigilin and regulates stress granule formation. Mol. Cancer Res. 19 (8), 1389–1397. 10.1158/1541-7786.MCR-20-1046 33888601

[B81] Krueger D. A. Care M. M. Holland K. Agricola K. Tudor C. Mangeshkar P. (2010). Everolimus for subependymal giant-cell astrocytomas in tuberous sclerosis. N. Engl. J. Med. 363, 1801–1811. 10.1056/NEJMoa1001671 21047224

[B82] Lai M. Zou W. Han Z. Zhou L. Qiu Z. Chen J. (2021). Tsc1 regulates tight junction independent of mTORC1. Proc. Natl. Acad. Sci. U. S. A 118 (30), e2020891118. 10.1073/pnas.2020891118 34301883 PMC8325158

[B83] Lee B. J. Mallya S. Dinglasan N. Fung A. Nguyen T. Herzog L. O. (2021). Efficacy of a novel bi-steric mTORC1 inhibitor in models of B-cell acute lymphoblastic leukemia. Front. Oncol. 11, 673213. 10.3389/fonc.2021.673213 34408976 PMC8366290

[B84] Lee D. F. Kuo H. P. Chen C. T. Hsu J. M. Chou C. K. Wei Y. (2007). IKK beta suppression of TSC1 links inflammation and tumor angiogenesis via the mTOR pathway. Cell. 130, 440–455. 10.1016/j.cell.2007.05.058 17693255

[B85] Li Y. Cao J. Chen M. Li J. Sun Y. Zhang Y. (2017). Abnormal neural progenitor cells differentiated from induced pluripotent stem cells partially mimicked development of TSC2 neurological abnormalities. Stem Cell. Rep. 8 (4), 883–893. 10.1016/j.stemcr.2017.02.020 PMC5390135 28344003

[B86] Liu M. Zeng T. Zhang X. Liu C. Wu Z. Yao L. (2018). ATR/Chk1 signaling induces autophagy through sumoylated RhoB-mediated lysosomal translocation of TSC2 after DNA damage. Nat. Commun. 9 (1), 4139. 10.1038/s41467-018-06556-9 30297842 PMC6175864

[B87] Lynham J. Houry W. A. (2022). The role of Hsp90-R2TP in macromolecular complex assembly and stabilization. Biomolecules 12, 1045. 10.3390/biom12081045 36008939 PMC9406135

[B88] Ma L. Chen Z. Erdjument-Bromage H. Tempst P. Pandolf P. P. (2005). Phosphorylation and functional inactivation of TSC2 by erk implications for tuberous sclerosis and cancer pathogenesis. Cell. 121, 179–193. 10.1016/j.cell.2005.02.031 15851026

[B89] MacKeigan J. P. Krueger D. A. (2015). Differentiating the mTOR inhibitors everolimus and sirolimus in the treatment of Tuberous sclerosis complex. Neuro Oncol. 17 (12), 1550–1559. 10.1093/neuonc/nov152 26289591 PMC4633932

[B90] Madigan J. P. Hou F. Ye L. Hu J. Dong A. Tempel W. (2018). The Tuberous sclerosis complex subunit TBC1D7 is stabilized by akt phosphorylation-mediated 14-3-3 binding. J. Biol. Chem. 293 (42), 16142–16159. 10.1074/jbc.RA118.003525 30143532 PMC6200923

[B91] Marshall C. B. Ho J. Buerger C. Plevin M. J. Li G. Y. Li Z. (2009). Characterization of the intrinsic and TSC2-GAP-regulated GTPase activity of rheb by real-time NMR. Sci. Signal 2 (55), ra3. 10.1126/scisignal.2000029 19176517

[B92] McEneaney L. J. Tee A. R. (2019). Finding a cure for Tuberous sclerosis complex: from genetics through to targeted drug therapies. Adv. Genet. 103, 91–118. 10.1016/bs.adgen.2018.11.003 30904097

[B93] Menon S. Dibble C. C. Talbott G. Hoxhaj G. Valvezan A. J. Takahashi H. (2014). Spatial control of the TSC complex integrates insulin and nutrient regulation of mTORC1 at the lysosome. Cell. 156, 771–785. 10.1016/j.cell.2013.11.049 24529379 PMC4030681

[B94] Metzker M. L. (2010). Sequencing technologies - the next generation. Nat. Rev. Genet. 11, 31–46. 10.1038/nrg2626 19997069

[B95] MietzschU.McKennaJ.ReithR. M.WayS. W.GambelloM. J. (2013). Comparative analysis of TSC1 and TSC2 single and double radial glial cell mutants. J. Comp. Neurol. 521, 3817–3831. 10.1002/cne.23380 23749404

[B96] Mingarelli A. Vignoli A. La Briola F. Peron A. Giordano L. Banderali G. (2018). Dramatic relapse of seizures after everolimus withdrawal. Eur. J. Paediatr. Neurol. 22 (1), 203–206. 10.1016/j.ejpn.2017.07.018 28888335

[B97] Mita M. M. Mita A. C. Chu Q. S. Rowinsky E. K. Fetterly G. J. Goldston M. (2008). Phase I trial of the novel Mammalian target of rapamycin inhibitor deforolimus (AP23573; MK-8669) administered intravenously daily for 5 days every 2 weeks to patients with advanced malignancies. J. Clin. Oncol. 26 (3), 361–367. 10.1200/JCO.2007.12.0345 18202410

[B98] Moon R. T. Kohn A. D. De Ferrari G. V. Kaykas A. (2004). WNT and beta-catenin signalling: diseases and therapies. Nat. Rev. Genet. 5 (9), 691–701. 10.1038/nrg1427 15372092

[B99] Morales Y. CaÃÅceres T. May K. Hevel J. M. (2016). Biochemistry and regulation of the protein arginine methyltransferases (PRMTs). Arch. Biochem. Biophys. 590, 138–152. 10.1016/j.abb.2015.11.030 26612103

[B100] Mozaffari M. Hoogeveen-Westerveld M. Kwiatkowski D. Sampson J. Ekong R. Povey S. (2009). Identification of a region required for TSC1 stability by functional analysis of TSC1missense mutations found in individuals with Tuberous sclerosis complex. BMC Med. Genet. 10, 88. 10.1186/1471-2350-10-88 19747374 PMC2753308

[B101] Nadadhur A. G. Alsaqati M. Gasparotto L. Cornelissen-Steijger P. van Hugte E. Dooves S. (2019). Neuron-glia interactions increase neuronal phenotypesin Tuberous sclerosis complex patient iPSC-derived models. Stem Cell. Rep. 12 (1), 42–56. 10.1016/j.stemcr.2018.11.019 PMC6335594 30581017

[B102] Nakashima A. Yoshino K. Miyamoto T. Eguchi S. Oshiro N. Kikkawa U. (2007). Identification of TBC7 having TBC domain as a novel binding protein to TSC1- TSC2 complex. Biochem. Biophys. Res. Commun. 361, 218–223. 10.1016/j.bbrc.2007.07.011 17658474

[B103] Nellist M. Brouwer R. W. Kockx C. E. van Veghel-Plandsoen M. Withagen-Hermans C. Prins-Bakker L. (2015). Targeted next generation sequencing reveals previously unidentified TSC1 and TSC2 mutations. BMC Med. Genet. 16, 10. 10.1186/s12881-015-0155-4 25927202 PMC4422413

[B104] Nellist M. Burgers P. C. van den Ouweland A. M. Halley D. J. Luider T. M. (2005a). Phosphorylation and binding partner analysis of the TSC1-TSC2 complex. Biochem. Biophys. Res. Commun. 333 (3), 818–826. 10.1016/j.bbrc.2005.05.175 15963462

[B105] Nellist M. Sancak O. Goedbloed M. A. Rohe C. van Netten D. Mayer K. (2005b). Distinct effects of single amino acid changes to tuberin on the function of the tuberin–hamartin complex. Eur. J. Hum. Genet. 13, 59–68. 10.1038/sj.ejhg.5201276 15483652

[B106] Northrup H. Aronow M. E. Bebin E. M. Bissler J. Darling T. N. de Vries P. J. (2021). Updated international Tuberous sclerosis complex diagnostic criteria and surveillance and management recommendations. Pediatr. Neurol. 123, 50–66. 10.1016/j.pediatrneurol.2021.07.011 34399110

[B107] Osborne J. P. Fryer A. Webb D. (1991). Epidemiology of tuberous sclerosis. Ann. N. Y. Acad. Sci. 615 (1), 125–127. 10.1111/j.1749-6632.1991.tb37754.x 2039137

[B108] Oyadomari S. Mori M. (2004). Roles of CHOP/GADD153 in endoplasmic reticulum stress. Cell. Death Differ. 11 (4), 381–389. 10.1038/sj.cdd.4401373 14685163

[B109] Prabhakar S. Cheah P. S. Zhang X. Zinter M. Gianatasio M. Hudry E. (2019). Long-term therapeutic efficacy of intravenous AAV-mediated hamartin replacement in mouse model of tuberous sclerosis type 1. Mol. Ther. Methods Clin. Dev. 15, 18–26. 10.1016/j.omtm.2019.08.003 31534984 PMC6745533

[B110] Prentzell M. T. Rehbein U. Cadena Sandoval M. De Meulemeester A. S. Baumeister R. Brohée L. (2021). G3BPs tether the TSC complex to lysosomes and suppress mTORC1 signaling. Cell. 184 (3), 655–674.e27. 10.1016/j.cell.2020.12.024 33497611 PMC7868890

[B111] Previtali R. Prontera G. Alfei E. Nespoli L. Masnada S. Veggiotti P. (2023). Paradigm shift in the treatment of tuberous sclerosis: effectiveness of everolimus. Pharmacol. Res. 195, 106884. 10.1016/j.phrs.2023.106884 37549757

[B112] Ramlaul K. Aylett C. H. S. (2018). Signal integration in the (m)TORC1 growth pathway. Front. Biol. (Beijing) 13 (4), 237–262. 10.1007/s11515-018-1501-7 32922443 PMC7116063

[B113] Ramlaul K. Fu W. Li H. de Martin Garrido N. He L. Trivedi M. (2021). Architecture of the tuberous sclerosis protein complex. J. Mol. Biol. 433 (2), 166743. 10.1016/j.jmb.2020.166743 33307091 PMC7840889

[B114] Randle S. C. (2017). Tuberous sclerosis complex: a review. Pediatr. Ann. 46 (4), e166–e171. 10.3928/19382359-20170320-01 28414398

[B115] Ranek M. J. Kokkonen-Simon K. M. Chen A. Dunkerly-Eyring B. L. Vera M. P. Oeing C. U. (2019). PKG1-modified TSC2 regulates mTORC1 activity to counter adverse cardiac stress. Nature 566, 264–269. 10.1038/s41586-019-0895-y 30700906 PMC6426636

[B116] Rayer P. F. O. (1835). Traits’ theorique et pratique des maladies de la peau. 2nd ed. Paris: JB Bailliere.

[B117] Roach E. S. (2016). Applying the lessons of tuberous sclerosis: the 2015 hower award lecture. Pediatr. Neurol. 63, 6–22. 10.1016/j.pediatrneurol.2016.07.003 27543366

[B118] Rodrik-Outmezguine V. S. Okaniwa M. Yao Z. Novotny C. J. McWhirter C. Banaji A. (2016). Overcoming mTOR resistance mutations with a new generation mTOR inhibitor. Nature 534, 272–276. 10.1038/nature17963 27279227 PMC4902179

[B119] Roux P. P. Ballif B. A. Anjum R. Gygi S. P. Blenis J. (2004). Tumor-promoting phorbol esters and activated ras inactivate the tuberous sclerosis tumor suppressor complex via p90 ribosomal S6 kinase. Proc. Natl. Acad. Sci. U. S. A. 101, 13489–13494. 10.1073/pnas.0405659101 15342917 PMC518784

[B120] Sancak O. Nellist M. Goedbloed M. Elfferich P. Wouters C. Maat-Kievit A. (2005). Mutational analysis of the TSC1 and TSC2 genes in a diagnostic setting: genotype – phenotype correlations and comparison of diagnostic DNA techniques in Tuberous sclerosis complex. Eur. J. Hum. Genet. 13, 731–741. 10.1038/sj.ejhg.5201402 15798777

[B121] Santiago Lima A. J. Hoogeveen-Westerveld M. Nakashima A. Maat-Kievit A. van den Ouweland A. Halley D. (2014). Identification of regions critical for the integrity of the TSC1-TSC2-TBC1D7 complex. PLoS ONE 9 (4), e93940. 10.1371/journal.pone.0093940 24714658 PMC3979717

[B122] Schönthal A. H. (2012). Endoplasmic reticulum stress: its role in disease and novel prospects for therapy. Sci. (Clairo) 2012, 857516. 10.6064/2012/857516 PMC3820435 24278747

[B123] Schonthal A. H. (2012). Targeting endoplasmic reticulum stress for cancer therapy. Front. Biosci. Sch. Ed. 4, 412–431. 10.2741/276 22202068

[B124] Schrötter S. Yuskaitis C. J. MacArthur M. R. Mitchell S. J. Hosios A. M. Osipovich M. (2022). The non-essential TSC complex component TBC1D7 restricts tissue mTORC1 signaling and brain and neuron growth. Cell. Rep. 39 (7), 110824. 10.1016/j.celrep.2022.110824 35584673 PMC9175135

[B125] Shohat G. Shani G. Eisenstein M. Kimchi A. (2002). The DAP-Kinase family of proteins: study of a novel group of calcium-regulated death-promoting kinases. Biochim. Biophys. Acta 1600, 45–50. 10.1016/s1570-9639(02)00443-0 12445458

[B126] Siroky B. J. Yin H. Babcock J. T. Lu L. Hellmann A. R. Dixon B. P. (2012). Human TSC-Associated renal angiomyolipoma cells are hypersensitive to ER stress. Am. J. Physiol. Ren. Physiol. 303 (6), F831–F844. 10.1152/ajprenal.00441.2011 PMC3468523 22791333

[B127] Stenson P. D. Mort M. Ball E. V. Evans K. Hayden M. Heywood S. (2017). The human gene mutation database: towards a comprehensive repository of inherited mutation data for medical research, genetic diagnosis and next-generation sequencing studies. Hum. Genet. 136, 665–677. 10.1007/s00439-017-1779-6 28349240 PMC5429360

[B128] Stevens C. Lin Y. Harrison B. Burch L. Ridgway R. A. Sansom O. (2009). Peptide combinatorial libraries identify TSC2 as a death-associated protein kinase (DAPK) death domain-binding protein and reveal a stimulatory role for DAPK in mTORC1 signaling. J. Biol. Chem. 284 (1), 334–344. 10.1074/jbc.M805165200 18974095

[B129] Stuart C. Fladrowski C. Flinn J. Öberg B. Peron A. Rozenberg M. (2021). Beyond the guidelines: how we can improve healthcare for people with Tuberous sclerosis complex around the world. Pediatr. Neurol. 123, 77–84. 10.1016/j.pediatrneurol.2021.07.010 34416612

[B130] Sullivan E. J. (1998). Lymphangioleiomyomatosis: a review. Chest 114, 1689–1703. 10.1378/chest.114.6.1689 9872207

[B131] Sweeney H. L. Houdusse A. (2010). Myosin VI rewrites the rules for myosin motors. Cell. 141 (4), 573–582. 10.1016/j.cell.2010.04.028 20478251

[B132] Taipale M. Tucker G. Peng J. Krykbaeva I. Lin Z. Y. Larsen B. (2014). A quantitative chaperone interaction network reveals the architecture of cellular protein homeostasis pathways. Cell. 158, 434–448. 10.1016/j.cell.2014.05.039 25036637 PMC4104544

[B133] Tate J. G. Bamford S. Jubb H. C. Sondka Z. Beare D. M. Bindal N. (2019). COSMIC: the catalogue of somatic mutations in cancer. Nucleic Acids Res. 47, D941–D947. 10.1093/nar/gky1015 30371878 PMC6323903

[B134] Teng J. M. Cowen E. W. Wataya-Kaneda M. Gosnell E. S. Witman P. M. Hebert A. A. (2014). Dermatologic and dental aspects of the 2012 international Tuberous sclerosis complex consensus statements. JAMA Dermatol 150, 1095–1101. 10.1001/jamadermatol.2014.938 25029267 PMC11100257

[B135] Thien A. Prentzell M. T. Holzwarth B. Kläsener K. Kuper I. Boehlke C. (2015). TSC1 activates TGF-β-Smad2/3 signaling in growth arrest and epithelial-to-mesenchymal transition. Dev. Cell. 32 (5), 617–630. 10.1016/j.devcel.2015.01.026 25727005

[B136] Tran L. H. Zupanc M. L. (2015). Long-term everolimus treatment in individuals with Tuberous sclerosis complex: a review of the current literature. Pediatr. Neurol. 53, 23–30. 10.1016/j.pediatrneurol.2014.10.024 26092412

[B137] Tyburczy M. E. Dies K. A. Glass J. Camposano S. Chekaluk Y. Thorner A. R. (2015). Mosaic and intronic mutations in TSC1/TSC2 explain the majority of TSC patients with no mutation identified by conventional testing. PLoS Genet. 11 (11), e1005637. 10.1371/journal.pgen.1005637 26540169 PMC4634999

[B138] Van Duyne G. D. Standaert R. F. Karplus P. A. Schreiber S. L. Clardy J. (1993). Atomic structures of the human immunophilin FKBP-12 complexes with FK506 and rapamycin. J. Mol. Biol. 229 (1), 105–124. 10.1006/jmbi.1993.1012 7678431

[B139] van Slegtenhorst M. de Hoogt R. Hermans C. Nellist M. Janssen B. Verhoef S. (1997). Identification of the tuberous sclerosis gene TSC1 on chromosome 9q34. Science 277 (5327), 805–808. 10.1126/science.277.5327.805 9242607

[B140] Vogt H. (1908). Zur Pathologie und pathologischen Anatomie der verschiedenen Idiotieform. Monatsschr Psychiatr. Neurol. 24, 106–150.

[B141] von Ranke F. M. Zanetti G. e Silva J. L. Araujo Neto C. A. Godoy M. C. Souza C. A. (2015). Tuberous sclerosis complex: State-Of-The-Art review with a focus on pulmonary involvement. Lung 193, 619–627. 10.1007/s00408-015-9750-6 26104489

[B142] von Recklinghausen F. (1862). Ein Herz von einem Neugeborene welches mehrere theils nach aussen. theils nach den Hohlen prominirende Tumoren (Myomen) trug. Monatsschr Geburtsheilk 20, 1–2.

[B143] Wang L. Zhou K. Fu Z. Yu D. Huang H. Zang X. (2017). Brain development and akt signaling: the crossroads of signaling pathway and neurodevelopmental diseases. J. Mol. Neurosci. 61, 379–384. 10.1007/s12031-016-0872-y 28025777 PMC5344939

[B144] Wang P. Sarkar S. Zhang M. Xiao T. Kong F. Zhang Z. (2024). DYRK1A interacts with the Tuberous sclerosis complex and promotes mTORC1 activity. eLife 12, RP88318. 10.7554/eLife.88318 39436397 PMC11495841

[B145] Wei S. Dai M. Zhang C. Teng K. Wang F. Li H. (2021b). KIF2C: a novel link between Wnt/Œ≤-catenin and mTORC1 signaling in the pathogenesis of hepatocellular carcinoma. Protein Cell. 12 (10), 788–809. 10.1007/s13238-020-00766-y 32748349 PMC8464548

[B146] Wei Z. Li P. He R. Liu H. Liu N. Xia Y. (2021a). DAPK1 (death associated protein kinase 1) mediates mTORC1 activation and antiviral activities in CD8+ T cells. Cell. Mol. Immunol. 18, 138–149. 10.1038/s41423-019-0293-2 31541182 PMC7852660

[B147] Wentink M. Nellist M. Hoogeveen-Westerveld M. Zonnenberg B. van der Kolk D. van Essen T. (2012). Functional characterization of the TSC2 c.3598C>T (p.R1200W) missense mutation that co-segregates with Tuberous sclerosis complex in mildly affected kindreds. Clin. Genet. 81 (5), 453–461. 10.1111/j.1399-0004.2011.01648.x 21332470

[B148] Woo H. H. Yi X. Lamb T. Menzl I. Baker T. Shapiro D. J. (2011). Posttranscriptional suppression of proto-oncogene c-fms expression by vigilin in breast cancer. Mol. Cell. Biol. 31, 215–225. 10.1128/MCB.01031-10 20974809 PMC3019847

[B149] Woodford M. R. Sager R. A. Marris E. Dunn D. M. Blanden A. R. Murphy R. L. (2017). Tumor suppressor Tsc1 is a new Hsp90 co-chaperone that facilitates folding of kinase and non-kinase clients. EMBO J. 36 (24), 3650–3665. 10.15252/embj.201796700 29127155 PMC5730846

[B150] Yang B. Li S. Wang H. Guo Y. Gessler D. J. Cao C. (2014). Global CNS transduction of adultmice by intravenously delivered rAAVrh.8 and rAAVrh.10 and nonhuman Primates byrAAVrh.10. Mol. Ther. 22, 1299–1309. 10.1038/mt.2014.68 24781136 PMC4089005

[B151] Yang H. Yu Z. Chen X. Li J. Li N. Cheng J. (2021). Structural insights into TSC complex assembly and GAP activity on rheb. Nat. Commun. 12, 339. 10.1038/s41467-020-20522-4 33436626 PMC7804450

[B152] Zhan J. Chitta R. K. Harwood F. C. Grosveld G. C. (2019). Phosphorylation of TSC2 by PKC-δ reveals a novel signaling pathway that couples protein synthesis to mTORC1 activity. Mol. Cell. Biochem. 456 (1-2), 123–134. 10.1007/s11010-019-03498-8 30684133

[B153] Zhang J. Kim J. Alexander A. Cai S. Tripathi D. N. Dere R. (2013). A Tuberous sclerosis complex signalling node at the peroxisome regulates mTORC1 and autophagy in response to ROS. Nat. Cell. Biol. 15, 1186–1196. 10.1038/ncb2822 23955302 PMC3789865

