# Supplementary material for: New insights into tuberous sclerosis complex: from structure to pathogenesis
Source: Front Cell Dev Biol. 2025 Jun 27;13:1595867. doi: 10.3389/fcell.2025.1595867 (PMC12245910; doi:10.3389/fcell.2025.1595867)
Supplement: Supplementary file 1 [file DataSheet1.pdf]

Supplementary Table 1. TSC2 phosphorylation sites and corresponding kinases

Kinase	TSC2 phosphorylation sites	mTORC1 activities
AKT	S939, S981, S1130, S1132, T1464	+
AMPK	T1271, S1383, S1387	-
DAPK1	S939	+
DYRK1A	T1464	+
ERK1/2	S540, S664	+
GSK-3β	S1337, S1341	-
PKC-δ	S932, S939	+
PKG1	S1364, S1365	+
RSK1	S1364, S1798	+
+ upregulated; - downregulated		

Supplementary Table 2. Classification of pathogenic TSC1 and TSC2 variants

Classification of variants	TSC1	TSC2
Frameshift	543	1054
Premature termination codon	195	349
Splicing/ unknown issues	129	970
Missense	50	571
Total	917	2944

All the TSC1 and TSC2 variants are from TSC Leiden Open Variant Databases (https://databases.lovd.nl/shared/genes?search_name=tuberous%20sclerosis). These unique DNA variants are clinically classified as either pathogenic or likely pathogenic.

Supplementary Table 3. Structural classification of TSC2 variants.

TSC2 Variant	Predicted structural effect	Functional assay
P91L	Disrupts hydrophobic packing	Active (1)
R98W	Disrupts polar interactions	N.D.
L105P	Breaks helix	N.D.
L180P	Breaks helix	N.D.
L219P	Breaks helix	N.D.
C244R	Affects packing	Inactive (2)
M286V	None	N.D.
G300V	Breaks helix	N.D.
W304R	Disrupts hydrophobic packing	N.D.
E337K	Disrupts polar interactions	N.D.
L340P	Breaks helix	Inactive (2)
K347R	None	N.D.
A357V	Affects packing; close to TSC1/TSC2 interface	Inactive (2)
R367Q	None	Active (2 & 3)
Y407D	Disrupts polar interactions	N.D.
L410R	Disrupts hydrophobic packing	Inactive (1)
P419S	Breaks turn	Inactive (2)
A431V	Affects packing	N.D.
L448P	Breaks helix	Inactive (1)
A460T	None	Active (2)
R462H	Breaks turn	Active (2)
L493V	Affects packing	Inactive (1)
L493P	Breaks turn	Inactive (1)
V504D	Affects packing	N.D.
N525S	None	Active (2 & 3)
K533N	None	N.D.
R585H	Breaks helix	N.D.
H597R	Disrupts polar interactions	N.D.
H597Y	Affects packing	Inactive (1)
Y598H	Disrupts polar interactions	Inactive (2)
Y598C	Disrupts polar interactions	N.D.
A607T	Breaks turn	Active (2)
R611Q	Disrupts polar interactions	Inactive (3)
R611W	Disrupts polar interactions	Inactive (3)
R611G	Disrupts polar interactions	Inactive (3)
F615S	Disrupts hydrophobic packing	Weak (3)
L618P	Breaks helix	N.D.
R622W	Breaks strand	Inactive (2)
R622P	Breaks strand	N.D.
D647N	None	N.D.
M649T	None	N.D.
M649L	None	N.D.
A678T	None	N.D.

L693P	Breaks helix	Active (2)
C696Y	Disrupts hydrophobic packing	Inactive (2 & 3)
C696R	Disrupts hydrophobic packing	Inactive (3)
E700K	Disrupts polar interactions	N.D.
V705M	Affects packing	Inactive (1)
L737P	Breaks helix	N.D.
L792R	Disrupts hydrophobic packing	N.D.
C804R	Disrupts hydrophobic packing	Inactive (2)
L808S	Disrupts hydrophobic packing	N.D.
L826M	Disrupts hydrophobic packing	Active (2)
L826P	Breaks helix	Active (3)
L830P	Breaks helix	N.D.
L847P	Breaks helix	N.D.
P878S	Breaks turn	N.D.
F897S	Disrupts hydrophobic packing	Inactive (2)
R905W	Breaks helix; close to TSC2 dimerisation interface	N.D.
R905Q	Breaks helix; close to TSC2 dimerisation interface	Inactive (2 & 3)
L916P	Disrupts hydrophobic packing	N.D.
L916R	Breaks helix	Inactive (2)
L1027P	Breaks helix; close to TSC2 dimerisation interface	N.D.
D1028N	Disrupts polar interactions; close to TSC2 dimerisation interface	Inactive (2)
M1030R	Disrupts hydrophobic packing; close to TSC2 dimerisation interface	N.D.
R1032P	Breaks helix; close to TSC2 dimerisation interface	Inactive (2)
L1061P	Breaks strand; close to TSC2 dimerisation interface and GAP domain	Inactive (2)
T1068I	Break strand; close to TSC2 dimerisation interface and GAP domain	Inactive (2)
Y1186C	Disrupts hydrophobic packing	N.D.
R1200W	Disrupts polar interactions; close to TSC2 dimerisation interface and GAP domain	Inactive (4)
R1200P	Breaks strand; close to TSC2 dimerisation interface and GAP domain	N.D.
T1203P	Disrupts polar interactions; close to TSC2 dimerisation interface and GAP domain	N.D.

G1204E	Affects packing; close to TSC2 dimerisation interface and GAP domain	Inactive (2)
P1497R	Breaks helix; close to TSC2 dimerisation interface and GAP domain	N.D.
P1497L	Breaks helix; close to TSC2 dimerisation interface and GAP domain	N.D.
P1497S	Breaks helix; close to TSC2 dimerisation interface and GAP domain	N.D.
P1497T	Breaks helix; close to TSC2 dimerisation interface and GAP domain	N.D.
S1498N	Disrupts hydrophobic packing; close to TSC2 dimerisation interface and GAP domain	N.D.
S1498T	Disrupts hydrophobic packing; close to TSC2 dimerisation interface and GAP domain	N.D.
Q1503P	Breaks helix and disrupts polar interactions; close to TSC2 dimerisation interface and GAP domain	Inactive (1)
D1535A	Disrupts polar interactions; close to TSC2 dimerisation interface and GAP domain	N.D.
L1548P	Break strand; close to TSC2 GAP domain	Inactive (3)
Y1549C	Disrupts polar interactions; close to TSC1 interface	Inactive (5)
Q1554H	Disrupts polar interactions; close to TSC1 interface	Inactive (2)
E1558K	Disrupts polar interactions	Inactive (5)
L1562P	Break helix	Inactive (5)
G1567D	Disrupts hydrophobic packing; close to TSC1 interface	Inactive (1)
Y1571N	Disrupts hydrophobic packing; close to TSC1 interface	Inactive (1)
L1578P	Breaks helix; close to TSC1 interface and TSC dimerization interface	Inactive (1)
H1620W	Disrupts polar interactions	N.D.
H1620R	Affects packing	Inactive (1)
T1623I	Disrupts hydrophobic packing; close to TSC1 interface	Inactive (1)

H1640Y	Disrupts polar interactions; putative Rheb-TSC2 interface	Inactive (5)
G1642D	Affects packing; putative Rheb-TSC2 interface	Inactive (2)
N1643H	Loss of active site residue; putative Rheb-TSC2 interface	Inactive (2)
N1643S	Loss of active site residue; putative Rheb-TSC2 interface	Inactive (2)
V1646M	Disrupts hydrophobic packing	Inactive (2)
V1646G	Disrupts hydrophobic packing	Inactive (2)
I1648T	Disrupts hydrophobic packing	Inactive (5)
Y1650C	Disrupts hydrophobic packing; close to TSC1 interface	N.D.
N1651S	Disrupts polar interactions; close to TSC1 interface	N.D.
P1673L	Disrupts hydrophobic packing	N.D.
P1675L	Breaks turn; close to TSC1 interface	Inactive (1)
P1675R	Breaks turn; close to TSC1 interface	N.D.
N1681K	Disrupts polar interactions	Inactive (5)
L1682P	Breaks strand	N.D.
S1684P	Affects packing	N.D.
P1709L	Breaks helix	Inactive (1)
V1711M	Affects packing	Inactive (1)
R1713H	Disrupts hydrophobic packing	Inactive (2)
R1713P	Breaks helix	Inactive (2)
L1717P	Breaks helix	N.D.
R1743Q	Disrupts polar interactions; TSC2 GAP domain	N.D.
R1743W	Disrupts polar interactions; TSC2 GAP domain	N.D.
R1743P	Disrupts polar interactions; TSC2 GAP domain	N.D.
R1743L	Disrupts polar interactions; TSC2 GAP domain	N.D.
E1756	Disrupts polar interactions	N.D.

TSC2 variants were reported at least 5 on TSC2 Leiden Open Variant Databases (LOVD; http:// http://www.lovd.nl/TSC2). Predicted structure effects are based on the mutagenesis stimulation using the published TSC complex model (PDB 9ce3).

1. Hoogeveen-Westerveld M, Wentink M, van den Heuvel D, Mozaffari M, Ekong R, et al. Functional assessment of variants in the TSC1 and TSC2 genes identified in individuals with tuberous sclerosis complex. Hum Mutat. (2011) 32(4):424-435.
2. Hoogeveen-Westerveld M, Ekong R, Povey S, Mayer K, Lannoy N, et al. Functional assessment of TSC2 variants identified in individuals with tuberous sclerosis complex. Hum Mutat. (2013) 34(1):167-175.
3. Sancak O, Nellist M, Goedbloed M, Elfferich P, Wouters C, et al. Mutational analysis of the TSC1 and TSC2 genes in a diagnostic setting: genotype – phenotype correlations and comparison of diagnostic DNA techniques in Tuberous Sclerosis Complex. Eur J Hum Genet (2005) 13:731–741.
4. Wentink M, Nellist M, Hoogeveen-Westerveld M, Zonnenberg B, van der Kolk D. et al. Functional characterization of the TSC2 c.3598C>T (p.R1200W) missense mutation that co-segregates with tuberous sclerosis complex in mildly affected kindreds. Clin Genet. 2012 May;81(5):453-461.
5. Hansmann P, Brückner A, Kiontke S, Berkenfeld B, Seebohm G, et al. Structure of the TSC2 GAP Domain: mechanistic insight into catalysis and pathogenic mutations. Structure. (2020) 28(8):933-942.e4.

Supplementary Table 4. Structural classification of TSC1 variants.

TSC1 variant	Predicted structural effect	Functional assay
K30R	None	N.D.
L50P	Breaks helix	Inactive (1 & 2)
E51D	None	Active (1 & 2)
L61P	Breaks helix	Inactive (1 & 2)
L64W	Disrupts hydrophobic packing	N.D.
A84T	None	N.D.
L116V	None	N.D.
K121R	Disrupts polar interactions	N.D.
V178I	None	N.D.
F188V	Disrupts hydrophobic packing	N.D.
R190C	Disrupts polar interactions	Inactive (1 & 2)
L191R	Disrupts hydrophobic packing	N.D.
P196L	Disrupts hydrophobic packing	N.D.
F216S	Disrupts hydrophobic packing; close to TSC1 dimerisation interface	N.D.
M224R	Disrupts hydrophobic packing; close to TSC1 dimerisation interface	N.D.
R246K	Disrupts polar interactions; close to TSC1 dimerisation interface	N.D.
Q654E	Disrupts polar interactions	N.D.
H732Y	Affects packing; close to TSC2 interface	N.D.
N762S	None	Active (3)
T899S	None	N.D.

TSC1 variants were reported at least 5 on TSC1 Leiden Open Variant Databases (LOVD; http:// www.lovd.nl/TSC1). Predicted structure effects are based on the mutagenesis stimulation using the published TSC complex model (PDB 9ce3).

1. Mozaffari M, Hoogeveen-Westerveld M, Kwiatkowski D, Sampson J, Ekong R, et al. Identification of a region required for TSC1 stability by functional analysis of TSC1missense mutations found in individuals with tuberous sclerosis complex. BMC Med Genet. (2009) 10:88.
2. Hoogeveen-Westerveld M, Wentink M, van den Heuvel D, Mozaffari M, Ekong R, et al. Functional assessment of variants in the TSC1 and TSC2 genes identified in individuals with tuberous sclerosis complex. Hum Mutat. (2011) 32(4):424-435.
3. Hoogeveen-Westerveld M, Ekong R, Povey S, Karbassi I, Batish SD, et al. Functional assessment of TSC1 missense variants identified in individuals with tuberous sclerosis complex. Hum Mutat. 2012 Mar;33(3):476-479.
